# The Comparative Method Based on Coronary Computed Tomography Angiography for Assessing the Hemodynamic Significance of Coronary Artery Stenosis

**DOI:** 10.1007/s13239-023-00658-2

**Published:** 2023-03-03

**Authors:** Zbigniew Małota, Wojciech Sadowski, Konrad Pieszko, Rafał Zimoląg, Filip Czekała, Renata Malinowska, Jarosław Hiczkiewicz

**Affiliations:** 1 Institute of Heart Prostheses, Professor Zbigniew Religa Foundation of Cardiac Surgery Development, Zabrze, Poland; 2 Department of Interventional Cardiology and Cardiac Surgery, Collegium Medicum, University of Zielona Góra, Góra, Poland; 3 Clinical Department of Cardiology, Multidisciplinary Hospital, Independent Public Healthcare Center in Nowa Sól, Nowa Sól, Poland

**Keywords:** Fractional flow reserve, Coronary artery stenosis, Computational fluid dynamics, Hemodynamic indices, Coronary computed tomography angiography, Flow energy

## Abstract

**Purpose:**

An important aspect in the prevention and treatment of coronary artery disease is the functional evaluation of narrowed blood vessels. Medical image-based Computational Fluid Dynamic methods are currently increasingly being used in the clinical setting for flow studies of cardio vascular system. The aim of our study was to confirm the feasibility and functionality of a non-invasive computational method providing information about hemodynamic significance of coronary stenosis.

**Methods:**

A comparative method was used to simulate the flow energy losses in real (stenotic) and reconstructed models without (reference) stenosis of the coronary arteries under stress test conditions, i.e. for maximum blood flow and minimal, constant vascular resistance.

In addition to the absolute pressure drop in the stenotic arteries (*FFR*_sten_) and in the reconstructed arteries (*FFR*_rec_), a new energy flow reference index (EFR) was also defined, which expresses the total pressure changes caused by stenosis in relation to the pressure changes in normal coronary arteries, which also allows a separate assessment of the haemodynamic significance of the atherosclerotic lesion itself. The article presents the results obtained from flow simulations in coronary arteries, reconstructed on the basis of 3D segmentation of cardiac CT images of 25 patients from retrospective data collection, with different degrees of stenoses and different areas of their occurrence.

**Results:**

The greater the degree of narrowing of the vessel, the greater drop of flow energy. Each parameter introduces an additional diagnostic value. In contrast to *FFR*_sten_, the EFR indices that are calculated on the basis of a comparison of stenosed and reconstructed models, are associated directly with localization, shape and geometry of stenosis only. Both *FFR*_sten_ and EFR showed very significant positive correlation (*P* < 0.0001) with coronary CT angiography–derived FFR, with a correlation coefficient of 0.8805 and 0.9011 respectively.

**Conclusion:**

The study presented promising results of non-invasive, comparative test to support of prevention of coronary disease and functional evaluation of stenosed vessels.

**Supplementary Information:**

The online version contains supplementary material available at 10.1007/s13239-023-00658-2.

## Background

Cardiovascular diseases remain an unresolved issue in both health and social aspects.^5^ Coronary artery disease (CAD) is most often associated with the narrowing or blockage of blood vessels. Therefore, an important aspect of the prediction and treatment of CAD is the functional evaluation of narrowed blood vessels, especially in the case of an moderate stenosis, when reduction of the diameter of the artery lumen is in the range from 50 to 70%. It is estimated that about half of these stenoses are hemodynamically significant, although they are often overlooked at revascularization.^26,31^ According to the most recent recommendations for management of chronic coronary syndromes^19^ non-invasive functional imaging or Coronary Computed Tomography Angiography (CCTA) is recommended as an initial test to diagnose CAD in symptomatic patients. In case when non-invasive tests suggest high risk of coronary events and revascularization is considered, or when non-invasive tests are inconclusive, invasive coronary angiogram with the measurement of fractional flow reserve (FFR) remains the ultimate method to assess the significance of coronary lesions.

Computational Fluid Dynamics (CFD) based on non-invasive medical imaging has been also increasingly used for hemodynamic analysis of narrowed arteries.^23,25,30^ The most promising technique is CT-derived fractional flow reserve (CT-FFR). The HeartFlow Inc. is a pioneer to use CFD combined with lumped parameter models to calculate coronary blood flow, pressure, and FFR based on routinely acquired CCTA datasets and 3D segmentation. HeartFlow FFR_CT_ analysis is currently the only commercially available CT-FFR technique with FDA (U.S. Food and Drug Administration) clearance.

Several other techniques (cFFR- Siemens Healthcare, CT-FFR—Canon Medical Systems) have also been developed in recent years to non-invasive assessment of the coronary stenosis significance.^33^ However, they are still in the research phase.

CFD modelling of the Coronary Artery Disease carries several challenges, which range from 3D segmentation of coronary arteries and mesh generation to time-consuming numerical simulations. The complex lumped models require the definition of many patient specific parameters describing peripheral resistance and blood vessel compliance. On the other hand, simplified mathematical models, zero-dimensional and one-dimensional models, based on simplified representations of the components of the cardiovascular system, neglect fundamental physical properties of the 3D flow across variable geometry (arch, bifurcation, stenosis). The proper development of a numerical model of coronary vessels, that would be completely consistent with clinically obtained data, is a great challenge for researchers and very often requires invasive measurements. The hemodynamic significance of coronary artery stenosis depends both on the degree of stenosis, the type of atherosclerotic plaque, its shape, location, and the patient's health status and hemodynamic conditions in the cardiovascular system.^4^

The aim of our study was to develop and confirm the feasibility and functionality of a non-invasive computational technique, that partially overcomes these limitations, provides a new diagnostic information and can help in the assessment of the haemodynamic significance of coronary stenosis.

The VCAST™ is virtual coronary test using CT image-based CFD, to compare the pressure losses in stenosed arteries with pressure losses in normal, hypothetical healthy, non-stenosed, coronary arteries, carried out under conditions of cardiac stress test.

The test allows to define, among others, a new parameter describing the relative changes of pressure in relation to a healthy vessel, as well as for an assessment of the haemodynamic significance of the atherosclerotic lesion itself. In addition, consideration of dynamic pressure, so far neglected in determining FFR and assessing the haemodynamic significance of coronary stenosis, may help in assessing plaque progression, especially plaque erosion.

## Methods

In comparison to other methods of assessing the importance of haemodynamic coronary stenosis based on numerical modelling and non-invasive medical imaging the VCAST is characterized by the following assumptions and a new approach:

- Comparative analysis of flow through stenosed vessel with flow through normal, reconstructed, hypothetically healthy vessel is used;
- A new parameter describing the relative changes in pressure compared to a healthy vessel and allowing for additional, separate assessment of the haemodynamic significance of the atherosclerotic lesion itself;
- Evaluation of the physiological importance of a stenosis involve the static and total pressure;
- The boundary and initial conditions and distributions both of flow rate and pressure at inlet and outlet of coronary branches are specified on the basis of:
  - allometric laws,
  - pre-simulation of reconstructed, non-stenotic models;
- Numerical simulation is carried out for steady flow under cardiac stress test condition for which:
  - auto-regulation effect still retains the ability to maintain the flow rate with constant and minimal peripheral resistance. Resistance of each coronary branch of both models is calculated from maximum flow of reconstructed model and taking into account the zero-flow pressure,
  - the same values of flow rate are used for both models,
  - linear relationship between the distal pressure of the stenosed model and the pressure at the same location of the reconstructed mode (Online Appendix 1),

### VCAST Framework

**Figure 1:**
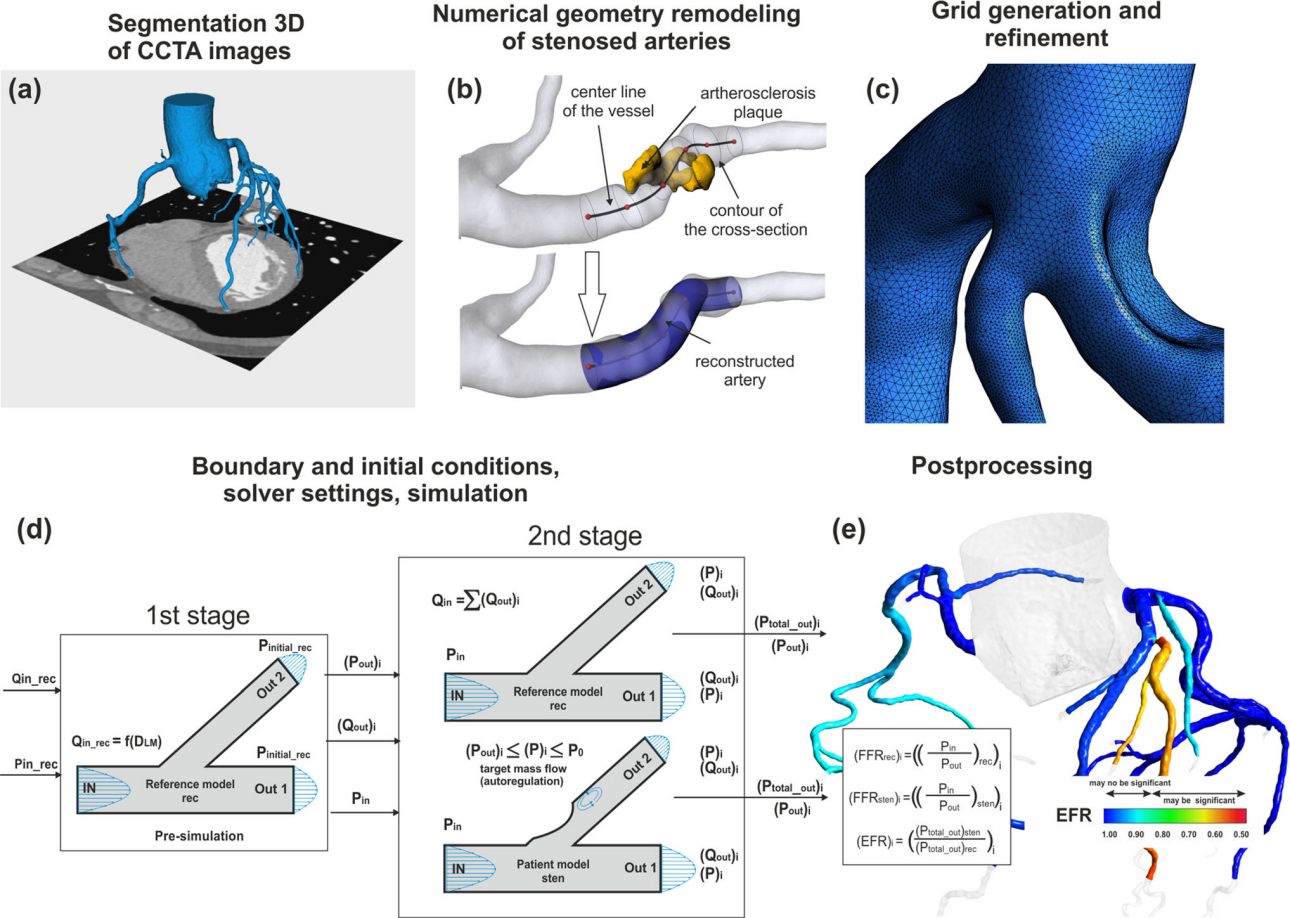
Stages of the VCAST™: (a) 3D segmentation of medical images, (b) numerical vessel reconstruction without stenosis based on analysis of cross-sectional area, centreline, and curvature of artery wall, (c) volume discretization, grid generation and refinement, (d) determination of initial and boundary conditions and simulation (e) the post-processing analysis, calculation of VCAST indices for all the branches of the models.

Schematic drawings illustrating the steps of VCAST is shown in Fig. 1. They include: patient specific segmentation of CCTA images, remodelling of stenosed model, discretization of 3D models, determine the initial and boundary condition, the solve the mass and momentum conservation equations to obtain the blood flow-pressure relation for both stenosis and reconstructed models, post processing, calculation of test indexes and results presentation.

During the test the two models of coronary vessel are created (Fig. 1):

- *The stenotic model* a model reflecting the actual state of the coronary vessels of the patient with existing atherosclerotic lesions. The geometry of this model is reproduced on the basis of CT images segmentation, in the DICOM format, by the 3DSlicer software packages.
- *The reference model* a model reflecting the hypothetical state of patient’s healthy vessels without stenoses. This model is made based on the numerical virtual reconstruction of the artery geometry and involves the removal of existing stenoses. The cross-sectional area of the vessel at the location of the stenosis is reproduced on the basis of approximation of the cross-sectional area before and after the stenosis. Reconstruction is performed in a 3D modelling software—Meshmixer (Autodesk Inc.).

### Patients and Vessel Characteristic

Since the purpose of the study is to pre-evaluate of the VCAST functionality, the studies were conducted on a small trial number. The article presents the results obtained from flow simulations in coronary arteries, reconstructed on the basis of 3D segmentation of cardiac CT images of 25 patients from retrospective data collection, with different degrees of stenoses and different areas of their occurrence.

Retrospective data were obtained in accordance with the medical procedures approved by the Collegium Medicum of the University of Zielona Góra and the Interdisciplinary Hospital in Nowa Sól.

Baseline patient and vessel characteristics are listed in Table 1.

**Table 1:** Baseline characteristic of the study population.

Characteristic	Mean	Min	Max	STD
No of patient	25			
Male	15 (60%)			
Age	63	40	82	9.8
Total calcium score, Agatston unit	339.11	64.3	776.5	272.58
Eject fraction [%]	71.93	57	86	7.62
Myocardial mass [g]	115.16	75	213	36.57
Cardiac output [l/min]	6.41	4.37	11.65	2.15
BMI	28.77	22.3	35.9	4.11
LM diameter [mm]	4.32	3.24	5.58	0.58
Stenosis [%]	57.08	33	80	12.40

All of the patients underwent invasive FFR measurement during routine angiography procedure no later than 3 months after the CT scan was obtained. The images from angiography were used to verify the geometry of coronary models, especially in place where plaques were localized.

Exclusion criteria included suspicion of acute coronary syndrome, prior coronary artery bypass graft (CABG), prior PCI, Body Mass Index > 35, intracoronary metallic stents, prior pacemaker or internal defibrillator lead implantation, prosthetic heart valves, significant arrhythmias or tachycardia that would preclude CT acquisition.

In the next studies, we plan to assess the diagnostic effectiveness of the VCAST on the basis of a larger sample size taking into account the statistical variability of the population, as well as the degree and distribution of coronary lesions.

### Numerical Implementation

The simulation was carried out in two stages:

- In the first stage, initial flow simulations was performed for the reference (reconstructed) model with predetermined boundary conditions corresponding to cardiac stress test conditions, i.e. for maximum blood flow and minimal, constant vascular resistance. Based on this pre-simulation of the reference model, compared to other CT FFR numerical methods, the patient-specific flow rate distribution at all coronary branches, was determined (instead of calculating from Murray's law).
- In the second stage, the flow was simulated with the boundary conditions calculated in the first stage (the inlet pressure and distribution of mass flow at outlets). We assumed that auto-regulation effect still had the ability to maintain the flow rate with minimal peripheral resistance. That way, the stenosed and reconstucted model was tested under the same haemodynamic conditions.

The patient-specific inlet flow conditions for first, preliminary stage were established based on morphometric flow-mass relationships in the heart obtained from direct measurements.^9^

These relationships show a direct correlation between flow rate and coronary inlet diameters:

1 $$Q_{in} = \beta D^{n}$$

wherein: *β*—correlation coefficient, *D*—diameter of inlet coronary artery, *n*—power number, *n* = 2.

The correlation coefficient of 0.5 has been determined for a statistically average diameter of 4.5 mm^12^ measured in the middle left main artery of right coronary artery-dominant in normal men and maximum value of flow rate during physical effort of 10 ml/s, over fivefold above resting values. In the first stage, the initial static pressure was determined at the level of 26 kPa, which corresponds to value of systolic blood pressure under dynamic exercise of high intensity condition.^14,16^

The pre-simulation of reconstructed non-stenotic model, in exercise condition, enables the determination of the correlation between the resistance of downstream vasculature of each coronary branch (*R*_*i*_), outflow (*Q*_*i*_) and pressure of the coronary tree. Taking into account the zero-flow pressure *P*_0_, i.e., the arterial blood pressure level at which flow stops,^13^ the resistance *R*_*i*_ can be defined as:

2 $$R_{i} = \frac{{P_{i} - P_{0} }}{{Q_{i} }}$$

The pressure at a pressure-outlet zone is adjusted in order to meet the desired mass flow rate by the targeted mass flow rate method which is based on the Bernoulli's equation.^3^

3 $${\text{d}}P = 0.5\rho \frac{{\left( {Q^{2} - Q_{{{\text{req}}}}^{2} } \right)}}{{\left( {\rho A} \right)^{2} }}$$

where: d*P*—change in pressure, *Q*—current computed mass flow rate at the pressure-outlet boundary, *Q*_req_—the required mass flow rate, *A*—area of the pressure-outlet boundary.

It should be noted, that clinical measurement of pressure during determination of FFR includes of zero-flow pressure. For this reason, we also took into consideration zero flow pressure (*P*_0_) of 2.8 kPa, as the lower limit of the outlet pressure (*P*_out_), derived from pressure-flow relationship during the normal heart cycle under vasodilatation conditions.^10,13,24^ However, some researchers believe,^10^ to provides a physiologically more accurate evaluation of the functional severity of a coronary artery stenosis, the values of FFR should not include the zero flow pressure.

### VCAST Indices

The mechanical energy flow rate EF expressed in Watt, which is needed to overcome the (constant) flow resistance and maintain the specified blood flow through cross sectional area of the coronary artery is represented as the product of the total pressure *P*_total_ and the mass flow rate *Q*^28^ (assuming no gravitational effects):

4 $$EF_{i} = \left( {\frac{1}{2}\rho u^{2} + P} \right)_{i} Q_{i} = (P_{{{\text{total}}}} )_{i} Q_{i} \left[ W \right]$$

Assuming the same inlet aortic flow energy (*EF*_a_) and the same flow rate distribution (*Q*_i_) at the outlet, for both normal (reconstructed) and stenotic models;

5 $$\left( {EF_{a} } \right)_{{{\text{rec}}}} = \left( {EF_{a} } \right)_{{{\text{sten}}}} = EF_{a} \quad \left( {Q_{i} } \right)_{{{\text{rec}}}} = \left( {Q_{i} } \right)_{{{\text{sten}}}} = Q_{i}$$

we will use three parameters to analyze the hemodynamic significance of the stenosis:

*FFR*_sten_—Fractional Flow Reserve for stenotic model- similarly to *FFR*_CT_ determines pressure drop in all places of the branch, calculated as the ratio of static pressure measured in stenotic model after the stenosis in the distal section of the vessel to the static pressure in the aorta:

6 $$FFR_{{{\text{sten}}}} = \left( {\frac{{P_{d} }}{{P_{a} }}} \right)_{{{\text{sten}}}}$$

*FFR*_rec_—Fractional Flow Reserve for reconstructed (references) model, determines the values of pressure drop in a healthy patient, calculated as the ratio of static pressure measured in reconstructed model after the stenosis in the distal section of the vessel to the static pressure in the aorta in a healthy patient:

7 $$FFR_{{{\text{rec}}}} = \left( {\frac{{P_{d} }}{{P_{a} }}} \right)_{{{\text{rec}}}}$$

EFR—Energy Flow Reference Index, defined as the ratio of energy losses in the branch of the stenotic model to the flow energy losses in the branch of the virtually reconstructed (references) coronary vessel without atherosclerotic lesions i.e. shows changes in relation to a healthy patient. The EFR can be calculated for both static and total pressure. For static pressure, the EFR express directly the ratio of *FFR*_sten_ to *FFR*_rec_. However, in the case of total pressure, EFR can be written as:

8 $$EFR = \frac{{\left( {\frac{{EF_{d} }}{{EF_{a} }}} \right)_{{{\text{sten}}}} }}{{\left( {\frac{{EF_{d} }}{{EF_{a} }}} \right)_{{{\text{rec}}}} }} = \frac{{\left( {EF_{d} } \right)_{{{\text{sten}}}} }}{{\left( {EF_{d} } \right)_{{{\text{rec}}}} }} = \frac{{\left( {P_{{{\text{total}}_d}} *Q_{i} } \right)_{sten} }}{{\left( {P_{{{\text{total}}_d}} *Q_{i} } \right)_{rec} }} = \frac{{\left( {P_{{{\text{total}}_d}} } \right)_{{{\text{sten}}}} }}{{\left( {P_{{{\text{total}}_d}} } \right)_{{{\text{rec}}}} }}$$

where: *P*_d_—average static pressure measured after the stenosis in the distal section of the vessel, *P*_a_—the average static pressure in the aorta, for both stenotic and reconstructed models, *P*_total_d_—total pressure measured in distal to stenosis for both stenotic and reconstructed models.

### Computational Model of Blood Flow

The commercial code FLUENT 19.2 (ANSYS-Fluent Inc.) was used to perform the numerical study. Coronary flow and pressure of blood in the coronary arteries were computed by solving the Navier–Stokes equations and the continuity equation for an incompressible fluid.^3^ The convergence criteria for the solver was set to 10^–5^ based on the root-mean-square of the residual at every node.

The *k*–*ω* with SST and low-Reynolds number turbulent model for flow of Newtonian fluid in rigid vessels, with a density of 1056 kg m^−3^ and a viscosity of 3.4 cP was applied (no-slip conditions, no heat transfer). The prediction of transition and turbulence in low-Re number flows is important when simulating the flow in stenosed blood vessels.

The same numerical model should be used for both the reconstructed model and the stenosed model. The model should reflect both laminar and transient flow conditions. In order to select an appropriate model for such a wide Reynolds number range that would correctly describe the complex flow, the laminar and selected turbulent models were compared with the experimental data of the FDA standard nozzle model (Online Appendix 2). Model selection and validation was made on the basis of the experimental pressure and particle image velocity (PIV) data of the standard FDA nozzle model (Online Appendix 3). In preliminary studies, the laminar model was compared with selected turbulent models for steady flow at Re = 500, 2000 and 3500. The simulation results clearly showed dependence on the kind of turbulence model, the place of measurement as well on and Re numbers. They are consistent with the results presented in the paper.^29^ The k-omega SST low Re model was finally selected, which ensured the assumed accuracy of calculation VCAST parameters in validation studies over the whole investigated range of Re (Online Appendix 2).

Due to the need to generate a large number of volumetric meshes, for both models related to the tested patients, the meshes were generated automatically by the script realized with the Ansys Fluent software.^3^ In order to properly resolve velocity gradients in the boundary layer, tetrahedral elements were refined in near-wall regions depending on the distance from the wall surface. The growth ratio of volumetric elements towards the vessel's center is 1.4. To mesh elements quality check, we used ANSYS Icem CFD software. The tetragonal mesh was selected on the basis of comparing the simulation results with the experimental results of the Nozzle model.

The mesh independence and refinement study was performed on a selected patient-specific model of left coronary artery with stenosis (~ 75%) in the proximal segment of the left circumflex (CX) branch. The mesh independence has been confirmed by the uncertainty study described in Online Appendix 3.

### Statistical Analysis

Fractional Flow Reserve (FFR) is currently the invasive gold standard diagnostic test for assessing the physiological significance of coronary arterial stenosis, confirmed by many clinical studies.

For this reason, it was used as a reference standard in correlation study with both *FFR*_sten_ and EFR for anonymized retrospective data (CCTA and angiography derived FFR).

The per-vessels results was analysed for the maximal stenoses identified in vessels, with the most adverse clinical status were selected to represent a given patient.

The Pearson correlation (with 95% confidence interval), evaluation of errors by Bland–Altman method and Specificity, Sensitivity, Positive Predictive Value (PPV), Negative Predictive Value (NPV) and accuracy were determined.

### Solution Verification and Numerical Model Validation

To solution verification and validate of numerical model was performed in accordance with ASME V&V 20-2009 Standard for Verification and Validation in Computational Fluid Dynamics and Heat Transfer.^11^ The Grid Convergence Index (GCI) method, based on Richardson extrapolation theory, was adopted for conducting the grid convergence study and for obtaining the numerical uncertainty due to discretization of the flow domain into finite volumes. The mesh refinement study was performed on a selected patient-specific model of left coronary artery with stenosis (~ 75%) in the proximal segment of the left circumflex (CX) branch. The control variables, characteristic parameters of VCAST (pressure, FFR_sten_, EFR) were calculated in two planes, normal to vessel axis, behind the stenosis in the circumflex (CX) and obtuse marginal (OM1) branches (Online Appendix 3).

The coarse, medium and fine tetragonal mesh with 651,013, 2,404,919 and 7,913,821 elements were generated using a constant value of refinement factor of ~ 1.5.

To validate the VCAST method, we used FDA nozzle model.^17^ This model reflects very well the hemodynamic properties of turbulent flow through the coronary stenosis. The simulation results were compared with experimental data of nozzle FDA model determined from an inter-laboratory particle image velocimetry (PIV) study, which are available in the data database;

https://ncihub.org/wiki/FDA_CFD/ComputationalRoundRobin1Nozzle.

The validation uncertainty combines the uncertainties due to numerical errors (*U*_num_), input parameters (*U*_inp_), and experimental measurements (*U*_exp_) and was calculated as:

9 $$U_{{{\text{val}}}} = \sqrt {U_{{{\text{num}}}}^{2} + U_{{{\text{inp}}}}^{2} + U_{{{\text{exp}}}}^{2} }$$

The *U*_val_ for FFR (Reynolds number 3500 and reference pressure 16,000 Pa) was of 0.021.

Additionally experiments on a hydraulic blood circulation system based on the Windkessel effect were performed. We compared the numerical solution with flow and pressure distribution in 3D printed, left coronary model with a 75% stenosis in the proximal LAD as well with the velocity profiles in the axially symmetrical model of 75% stenosis^21^ (Online Appendix 3).

## Results

### Velocity Distribution

**Figure 2:**
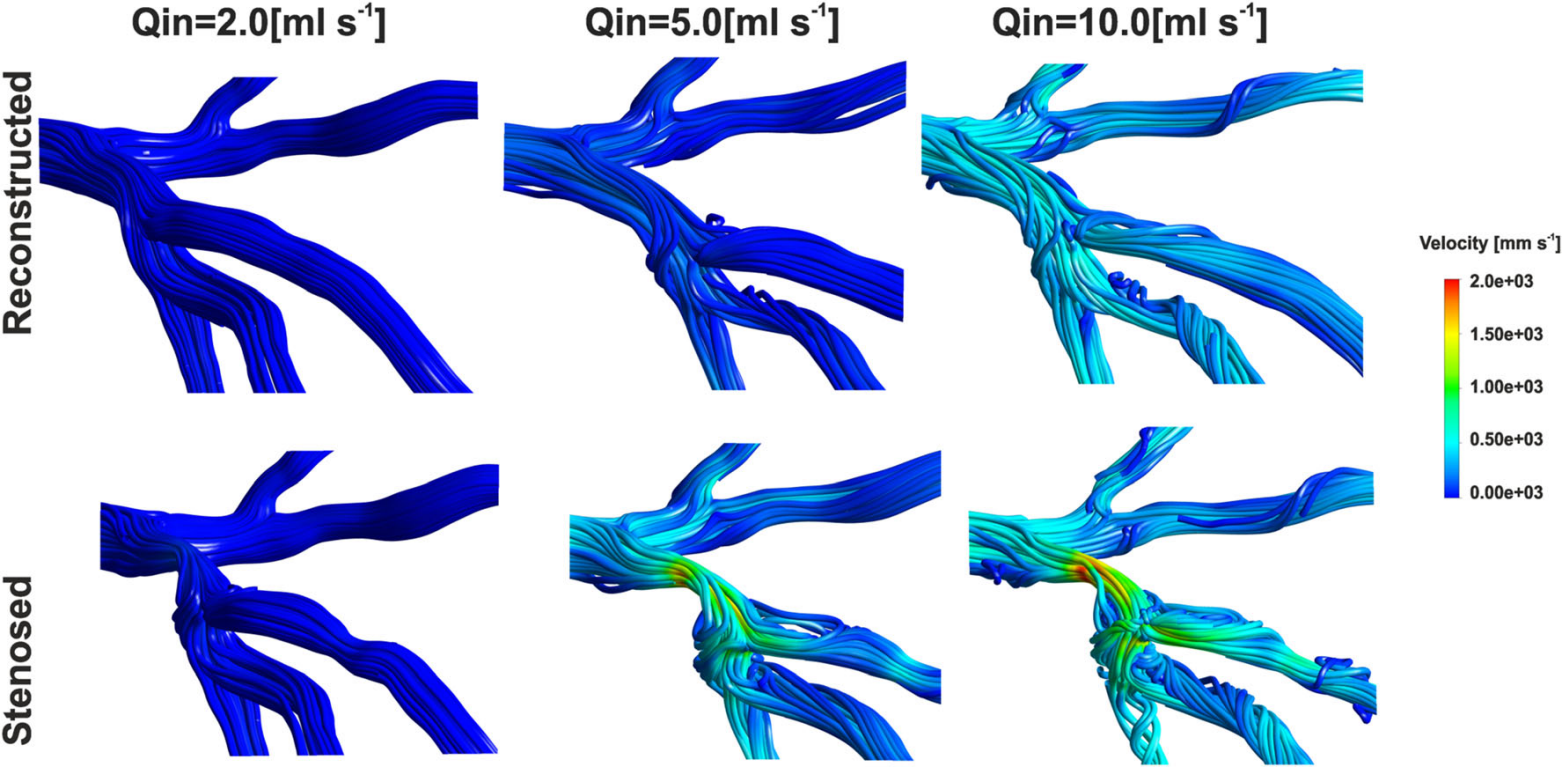
The flow disturbances in stenosed and reconstructed coronary vessel under rest (2.0 ml s^−1^), mild exercise (5.0 ml s^−1^) and high intensity exercise conditions (10 ml s^−1^).

Coronary artery stenosis causes significant flow disturbances in the area of stenosis (Fig. 2). In the constriction itself, the flow velocity and stress increases, and the pressure decreases. On the other hand, downstream to the narrowing, there is a separated swirl layer with negative flow which strongly depends on the shape and degree of stenosis.

For the flow under rest conditions (Fig. 2) with *Q*_*in*_ of 2 ml s^−1^, the blood flow is very orderly, parallel to the axis of vessel There are no eddies or swirls of fluid. Only very slight flow disturbances appear in the vessel branch downstream to stenosis. The nature of the flow is clearly laminar for both stenosed and reconstructed model. The greater the flow and the greater the degree of narrowing of the vessel, the greater the area of the flow disturbance is observed.

In the case of higher flow rate, with *Q*_*in*_ of 10 ml s^−1^, corresponding to the physical exercise conditions, significant flow disturbances appear causing an increase in energy loss during the flow, especially in the stenosed model. Narrowing just before branching also has an effect on flow disturbances in branches below the branching. Downstream to the narrowing, there is a negative flow in a separated swirl layer. The secondary flow is created and the flow stream in this branches is spiral.

**Figure 3:**
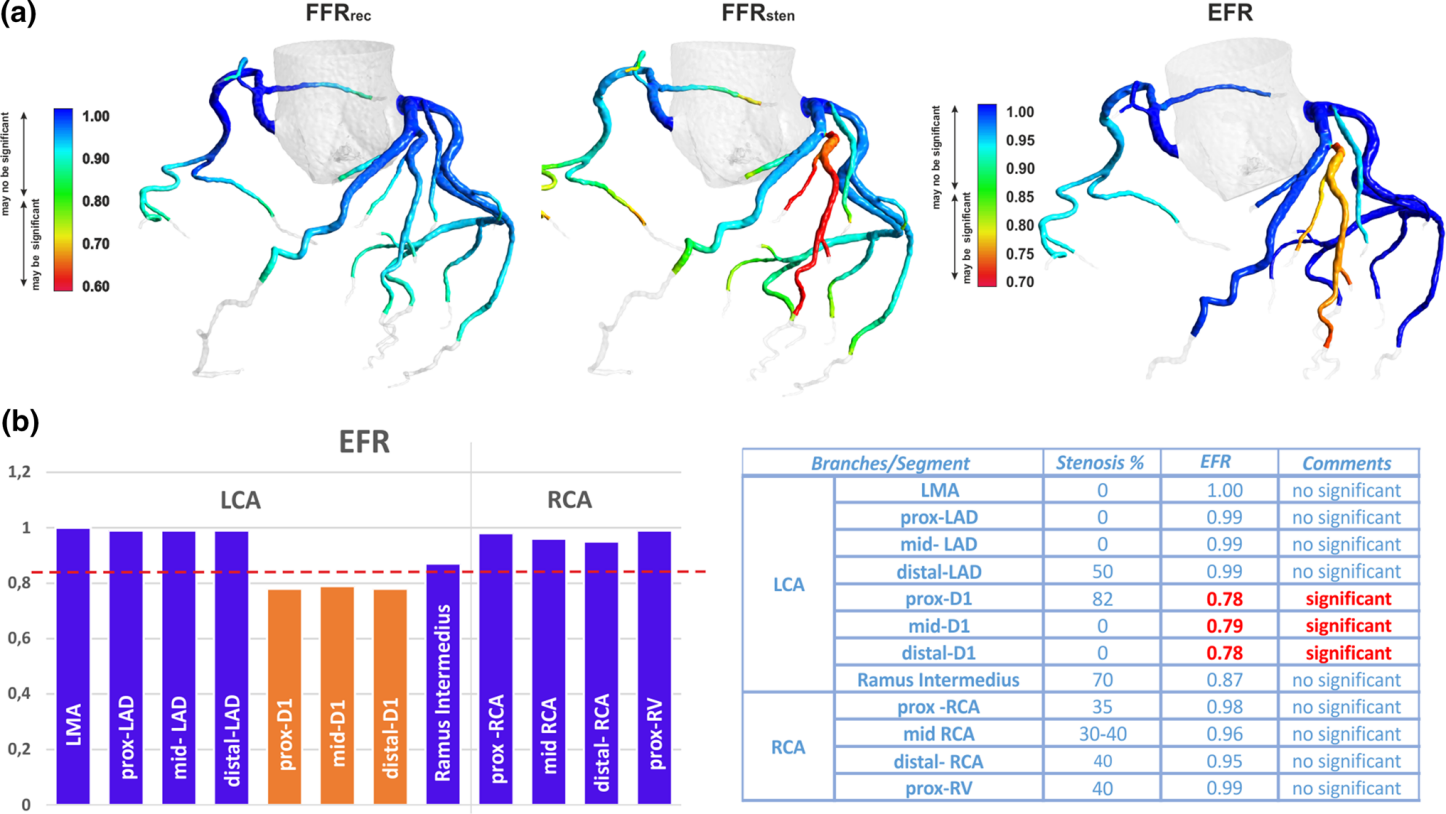
The example of VCAST™ results for severe stenosis in the proximal segment of D1 branches: (a) space distribution of FFRrec, FFRsten and EFR, (b) EFR results in the main segment of coronary arteries.

Figure 3 shows the example results of each of test indices distribution for a patient with a severe stenosis in proximal segment of D1.

### VCAST Indices

The *FFR*_rec_ for reconstructed model and *FFR*_*s*ten_ for stenotic model is determined as the ratio of the average pressure measured in the distal section of the vessel to the average pressure in the aorta. The EFR distribution calculated, as the ratio of the distal total pressure in the branch of the stenosed model to the distal total pressure in the branch of the reconstructed model. In all branches not directly affected by the stenosis, the EFR is close to one. In the D1 branch, the value of the EFR downstream to stenosis is less than 0.78. Unlike the *FFR*_rec_ and *FFR*_sten_, the distribution of the EFR is clearly independent of the vessel diameter.

**Figure 4:**
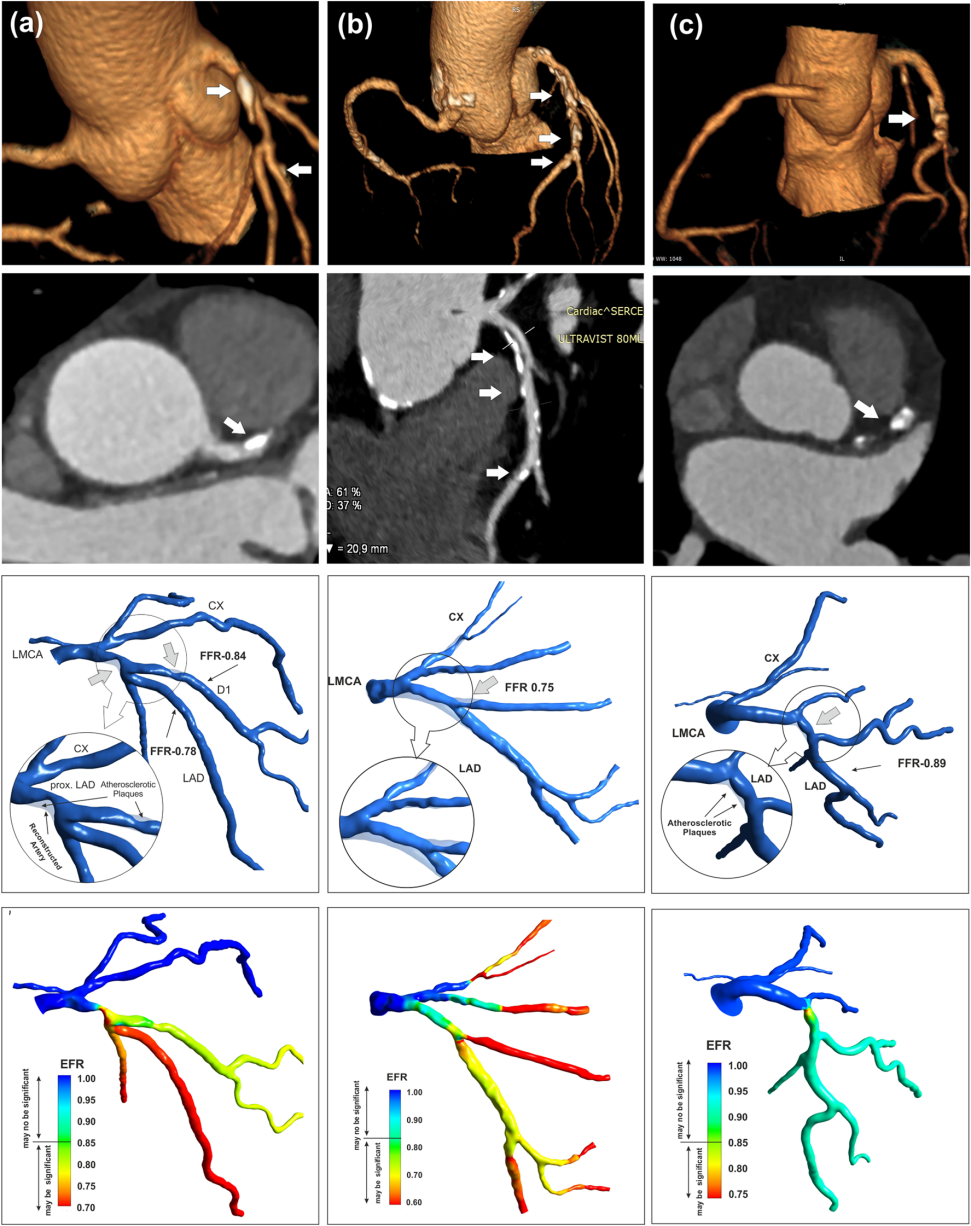
Example of EFR analysis of patient with different degrees of stenosis and different areas of their occurrence: (a) patient no. 2 with 60% narrowing and invasive FFR of 0.78 in proximal segment of LAD as well 50% narrowing and FFR of 0.84, measured in middle segment of diagonal branch D1, (b) of patient no. 2 with 60% narrowing and invasive FFR of 0.78 in proximal LAD as well 50% narrowing and invasive FFR of 0.84 in first diagonal branch, (c) of patient no. 22 with 60–70% narrowing and invasive FFR of 0.75 in proximal and middle LAD. From the top to bottom, respectively: volume rendering of LMCA, CT slice of normal and stenotic 3D models, EFR distribution in main branches.

The results were obtained for 25 randomly selected patients from retrospective data collection. Three selected studies of the left coronary artery with different degrees of stenoses and different areas of their occurrence are presented below on Fig. 4.

Figure 4a shows example of EFR distribution in left coronary arteries of patient with 60% narrowing and FFR of 0.78 in proximal segment of LAD as well 50% narrowing and FFR of 0.84, measured in middle segment of diagonal branch D1. These values of FFR corresponding to the EFR values of 0.70 and 0.86 respectively.

The effect of 70% diffuse stenosis in proximal and middle segment of LAD and in proximal segment of D1 on the EFR value is shown on Fig. 4b. EFR values below 0.85 confirms of the hemodynamic significance of stenosis. The EFR value of 0.79 in LAD downstream to the stenosis corresponds to the invasive FFR of 0.75 measured during coronary angiography. The significant stenosis were also found in the another vessels where the FFR measurement was not performed.

An example of EFR assessment for a vessel with no significant stenosis (*FFR* = 0.89) is shown in Fig. 4c. Stenosis of 52% in the middle segment of the LAD branch causes a drop in flow energy corresponding to an EFR value of 0.88.

### Statistical Analysis

**Figure 5:**
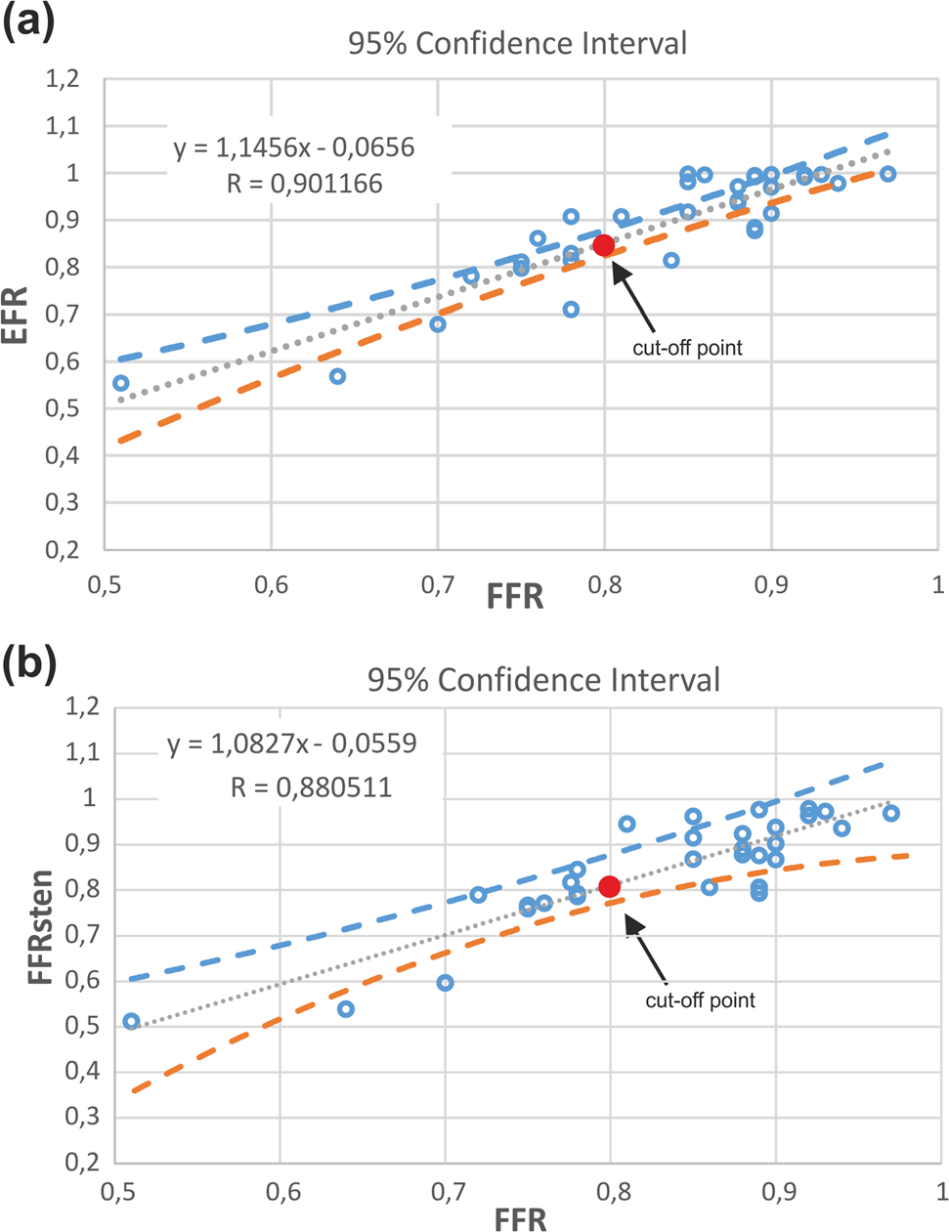
Correlation and 95% confidence interval plot between invasive FFR and: (a) EFR and (b) FFRsten.

The comparison of VCAST indices (FFR_sten_, EFR) with coronary CT angiography–derived FFR was assessed by correlation plot showing 95% confidence limits (Fig. 5). Both *FFR*_sten_ and EFR showed very significant positive correlation (*P* < 0.0001) with coronary CT angiography–derived FFR, with a correlation coefficient of 0.8805 and 0.9011 respectively.

**Figure 6:**
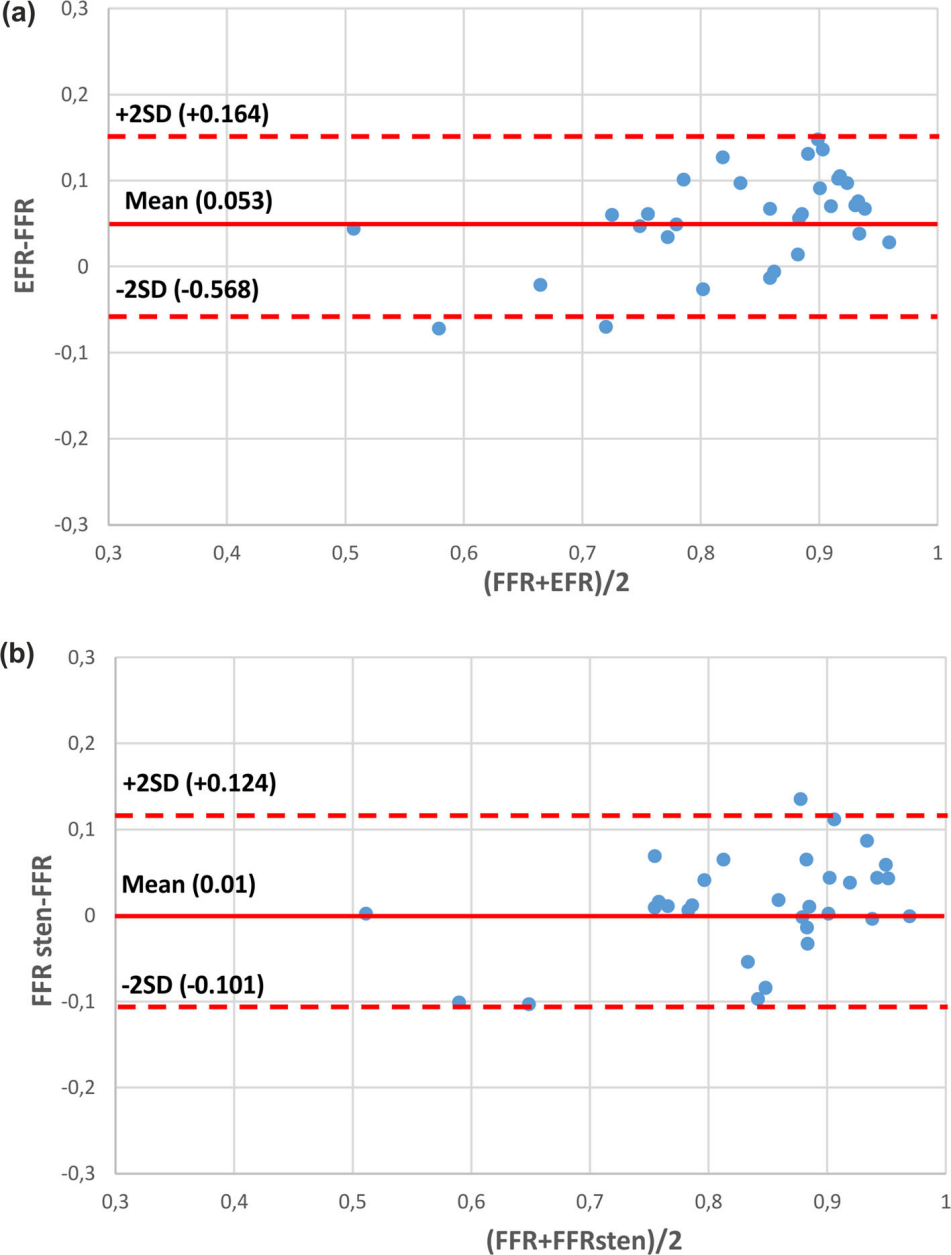
Bland–Altman plot comparing invasive FFR with (a) EFR and (b) FFRsten.

The mean value of the difference between both the FFR_sten_ and EFR with invasive FFR is presented on Bland–Altman plot (Fig. 6). Based on these results, we initially determined the cut-off points for FFR_sten_ and EFR of 0.81 ± 0.055 and 0.85 ± 0.053 respectively.

For the EFR, the overall specificity, sensitivity, positive predictive value (PPV), negative predictive value (NPV) were 95.2%, 81.8%, 90.0%, 90.9% and 90.6%, respectively.

### Solution Verification and Numerical Model Validation

The results of solution verification and numerical model validation are presented in Online Appendix 1.

The numerical uncertainty (*U*_num_) for the pressure, FFRsten and EFR downstream to the stenosis (plane 1 and plane 2) of 0.0132, 0.0137 and 0.0165 were calculated respectively.

The maximum value of Grid Convergence Index (GCI) and numerical uncertainty (*U*_num_) is 0.024 and 0.017 respectively.

A significant modeling error is observed for the pressure gradient, which results from a very large experimental pressure measurement error (Online Appendix 3) and input error.

However, for VCAST, the most important parameter on the basis of which the hemodynamic significance of stenosis is assessed is the parameter FFR. In this case the modeling error *δ*_model_ $\epsilon$ [− 0.0189, 0.0726] and [− 0.0161, 0.0241] for Re = 2000 and Re = 3500 respectively.

In case of experimental study, the error of FFR and EFR was calculated as mean differentials function of FFR = (*P*_i_/*P*_in_)_sten_ and EFR = *P*_i sten_/*P*_i rec_, respectively, where *P*_i_ is outlet pressure experimental of selected branches. Maximal error of VCAST indices is 8% (with the error of pressure measurement of 4%).

However, should be noted, the for VCAST test, very important is the set of particular flow rate on the selected outlet of branch. The flow rate in small coronary arteries is below 0.1 l/min. The maximum flow rate measurement error in this range is ± 0.035 l/min.

Therefore, our coronary model was made in a 2:1 scale. The set flow rates were twice as possible, according to criterion of the similarity of the Reynolds flow, which improved the flow rate accuracy. However, despite this we observed a difficulty in exact settings of coronary flow and keep the right flow balance sheet. The maximum difference between the inlet flow rate and the sum of the outlet flow rate, in the extreme case, was ~ 15%. This affect the measured pressure value because the pressure drop is a quadratic polynomial function of flow rate. More scaling of the model can significantly reduce this error.

In addition, lower pressure values were found in some branches without stenosis (Out 4-Out 6) of the stenosed model compared to the reconstructed model, as a result of which the experimental values of *FFR*_sten_ and EFR in these branches are lowered by about 3%.

## Discussion

The study of VCAST parameters provides an additional diagnostic values of stenosis significance in compare to FFR_CT_, cFFR or CT-FFR.

In addition to estimating the fractional flow reserve for both stenotic and reconstructed models, an Energy Flow Reference Index (EFR) is also calculated.

The EFR shows the relative changes in energy flow with respect to a healthy vessel, which enables a separate assessment of the haemodynamic significance of the atherosclerotic lesion itself.

The study of VCAST parameters provides an additional diagnostic values of stenosis significance in compare to FFRCT, cFFR or CT-FFR.

In addition to estimating the fractional flow reserve for both stenotic and reconstructed models, the Energy Flow Reference Index (EFR) is also calculated. EFR shows relative changes of energy flow in relation to a healthy vessel, which makes it possible a separate assessment of the haemodynamic significance of the atherosclerotic lesion itself.

FFR is clinical parameter based on the measurement of coronary pressures and just like the numerical estimated *FFR*_CT_ as well *FFR*_sten_ and *FFR*_rec_ parameters, takes into account both global and local pressure drops. Therefore, the pressure decreases with distance from the main artery not only in the presence of atherosclerotic lesions. The further the vessel is away from the main artery, the lower the value of FFR parameter. An FFR value below 0.8 does not always mean significant narrowing (Fig. 3a). It can also mean a distant small-diameter vessel. In contrast, the EFR parameters, due to differ local geometry of reconstructed and stenosed coronary models in the area of stenosis, are associated with pressure drop only near stenosis (Fig. 4). For this reason, the parameter shows only those places that result directly from atherosclerotic stenosis.

The fractional flow reserve FFR (*FFR*_sten_) 0.80 means that the static pressure drop downstream of the constriction is 20%, that results from both local and global losses. The decrease in total energy (total pressure) is less due to the pressure recovery phenomenon described above. On the other hand, the EFR value of 0.80 means a 20% local drop in total pressure caused only by stenosis.

The EFR, unlike FFR, more clearly separates significant stenosis from non-significant and its effect on the dynamics of blood flow in neighboring (downstream) coronary branches (Fig. 4a).

For normal arteries, without stenosis, these indices are close to 1. For all branches, where stenosis caused flow disturbances, this parameter take a value between 1 and 0. The greater the impact of stenosis on flow energy drop in any branch, the smaller index values.

However, it should be noted that, the assessment of the significance of the stricture only on the basis of the graphical presentation in the area of the stenosis and directly behind it in the place where there are large flow disturbances may be ambiguous. The assessment of a specific constriction should be done downstream to stenosis, in a place with as little disturbance of flow as possible, on the basis of the average value of the cross-sectional area normal to the vessel axis. This is the area where pressure measurement to determine the FFR parameters is the most suitable.

Therefore, the graphical 3D of the spatial distribution of the *FFR*_rec_, *FFR*_sten_ and *EFR*, reflects the values on the wall of the coronary vessel, while the results, that are presented in the VCAST report, in the form of a table (Fig. 3b) reflects the values of the indices, averaged over the cross-sectional area normal to the vessel axis, in specific places downstream to the stenosis, at a distance that will ensure undisturbed measurement conditions, i.e. where the flow is fully developed with a fixed boundary layer and a constant pressure drop.

The calculation of the indices downstream to stenosis is executed on the basis of the pressure, over the cross-sectional averaged area normal to the vessel axis. During the FFR_CT_ correlation analysis stage, the analysis is executed at the FFR invasive measurement site (determined on the basis of the position of the measuring catheter on the coronary angiography image).

However, in the case of clinical tests, the analysis will be performed at a distance that will ensure undisturbed measurement conditions, i.e. where the flow is fully developed with a fixed boundary layer and a constant pressure drop.

This is especially important in the case of multiple stenosis or near bifurcations. According to Casadonte *et al*.,^7^ in the case of local changes in vessel diameter by approximately 50%, multiple stenoses can be considered independent when the mutual distance between them exceeds six times the normal diameter of the vessel. When these changes are closer together, the energy flow drop is lower, due to limited energy diffusion (suppression of flow disturbance by the distal change). VCAS determines the actual energy losses induced by flow disturbances near single or multiple stenoses (sequential lesions). However, to avoid underestimating the significance of such complex stenosis, per- vessels stenosis with the maximal stenoses identified in vessels is analyses.

When blood flows through a constricted blood vessel, pressure and kinetic energy are converted (ignoring gravity), but some energy is lost mainly by friction. The energy losses take one of three forms: viscous losses, turbulence losses, and flow separation losses.^7,22^ Viscous energy losses are proportional to flow rate. Turbulent and seperated losses are in general proportional to the square of the flow rate.^1^ Turbulent losses usually do not occur in the healthy vessel but in severe stenosis they can be of greater magnitude than viscous losses. Additionally, the increased flow rate, during cardiac stress test, elicits additional blood flow disorders that are not present at rest.

However, it should be noted, there is a spatial offset along stenosis between the static pressure and the total pressure, that increases in magnitude with increased degree and length of stenosis. The greatest static pressure drop, caused by stenosis, is mainly observed in the initial region of stenosis, (contraction section), despite a small drop in total energy. Whereas, in the final area of the constriction, (expansion section) and immediately downstream to stenosis, a large amount of energy is lost, associated mainly with the losses of kinetic energy, despite, according to Bernoulli's equation, the increase in static pressure (pressure recovery phenomenon).

In addition to the haemodynamic assessment of the importance of coronary artery stenosis, a major problem is the assessment of plaque progression, in particular plaque erosion.

A large proportion of acute coronary events are the result of sudden vascular thrombosis due to erosion or rupture of the plaque. The accurate measurement of coronary stresses is still a challenge. Due to the complex shape of the vessels and the plaque itself, the description of the forces acting directly on the plaque, can be important additional information on the risk of plaque rupture and may help identify sensitive sites of stenoses. The main biomechanical factors affecting the susceptibility and mechanical instability of the atherosclerotic plaque are: its geometry, composition and hemodynamic stresses.^25^

In recent years, several publications confirmed the potential impact of axial forces induced by the flow stream, in atherosclerotic plaque rupture. (axial plaque stress -APS).^8^ The size of the APS is directly related to the dynamic pressure. For this reason, the additional kinetic energy analysis in the area of stenosis can also help in assessing the risk of atherosclerotic rupture, without the need to apply complex structural calculations.

Combined analysis of energy losses induced by viscous forces and momentum, close to stenosis, will allow to provide a more complete description of physiologic stenosis severity.^32^ For this reason, to analyze the hemodynamic significance of coronary stenosis, we used indices related to both static and total pressure drop.

Despite the use of a steady-state flow model, a high correlation of simulation results with clinical measurements was obtained.

Lo *et al*.,^20^ concluded that the flow pulsation, no significant effect on the calculated FFR in the narrowed coronary arteries. According to their research, the maximum difference of FFR between pulsatile and steady inflow conditions was 0.02 (2.4%).

The using numerical calculations for steady flow instead calculations for transient flow allowed for a significant reduction in the computation time from several hours to several minutes. For the acceleration of the execution time of test we used a high-performance computer (HPC). A total of 120 CPUs were used in parallel for the simulations. Average computational time for typical steady flow test of coronary arteries was approximately 540 s for one stages. However, due to still ongoing manual segmentation and reconstruction, the complete VCAST analyses for the patient, from downloading CT Images data to generating a final report for the doctor, currently takes several hours. This is comparable to the time taken to run the FFR_CT_ test, which use both the transient time-depended CFD and lumped parameters model. Once the segmentation and reconstruction time by machine learning algorithm based on deep, convolutional neural network, is reduced, our technique should be useful for assessing the hemodynamic significance of coronary artery stenosis.

Only methods based on 1D reduced-order models are characterized by a significantly shorter computational time. The 2nd generation of Siemens software reduced computational time to 4 min per patient. While, in case of Canon software, the time for edit of coronary arterial contour and centerline was 30–60 min, and the calculation time for CT-FFR was 20–30 min.^33^

However, it should be noted that despite the very high correlation between the 1D and 3D models, the 1D models lead to an overestimation of the FFR values, in particular for significant stenoses, for which the bias increase with degree of stenosis.^6^

The numerical analysis of pulsating blood flow when using complex lumped models (FFRCT) requires determination many patient-specific parameters and requires the use of high-performance computing. Additionally, cloud technology is used to upload CCTA images and download the FFRCT reports from the centralized core-laboratory.

The software of Siemens Healthcare (cFFR), and Canon Medical Systems (CT-FFR) are based on reduced-order modelling in combination with a lumped parameter model and a physiological model for the microvascular bed.^33^ In 2nd generation of cFFR the machine learning from CFD simulation on 12,000 synthetic coronary artery trees applied. Whereas, Canon Medical Systems (formerly Toshiba Medical) to estimate flow uses arterial lumen deformation in four diastolic phases assuming that resistance is constant and therefore pressure is proportional to flow.

Compared to other CT FFR numerical methods, the VCAST determine the flow velocity distribution based on the pre-simulation of the reference model, instead of calculating from Murray's law. There are deviations from Murray’s law, e.g. associated with a higher degree of calcification near coronary bifurcations, turbulent flow, as well with coronary artery anomalies. Therefore the application of Murray's law may lead to additional uncertainty in assessing the significance of the stenosis. The pre-simulation reduces the effect of these uncertainties.

A significant challenge posed by coronary flow modelling is also the fact that the coronary circulation has a high degree of autoregulation of coronary vascular resistance caused by coronary stenosis. Due to autoregulation coronary blood flow is maintained relatively constant over a range of perfusion pressures (~ 60–120 mmHg). Outside this range, among others in conditions of increased physical effort, flow becomes pressure-dependent and a loss of autoregulation is observed.^15^

The use of the same values of the flow rate in both models facilitates the correct determination of the autoregulation conditions of the coronary flow and allows to determine the decrease of the resistance in the model with the stenosis. Additionally, the use of the same flow rate distribution in both models, ensures that the observed differences in pressure drops in the stenosis and references model are the result of only the difference in geometry at the point of narrowing.

For this reason, the application of the comparative numerical simulation allows to minimize evaluation errors autoregulation and microcirculatory vasodilation that could lead to an incorrect assessment of the hemodynamic significance of coronary stenosis.

The different shapes of stenosis, the plaque size and lesion length, position of the stenosis, curvature and lumen diameter of artery, and boundary resistance has the significant impact on results of simulations and hemodynamic evaluation of significance of coronary artery stenosis.^15,18,27^ Even for stenosis with similar degree of stenosis, the value of EFR can be different due to the different shapes and localizations of stenosis.

## Limitation

There are several uncertainties and limitations to our study, that might lead to variations in the diagnostic indices and to misinterpretation, especially in the evaluation of functional severity of intermediate stenosis.

The objective of our research was to confirm the feasibility and functionality of a novel technique providing information about hemodynamic significance of coronary stenosis and not a clinical evaluation of the diagnostic value of the test.

Although the *FFR*_sten_ and EFR indexes have shown a high correlation with invasive FFR, this result was made on a very small clinical sample size and currently the clinical diagnostic value of the test cannot be fully confirmed. Consequently, despite the random selection of retrospective clinical data, the study of RCA is lacking and the VCAST results was obtained for the degree of stenosis in the range of 34% to 80% and FFR in the range of 0.51 to 0.97.

According to,^2^ for a sufficiently large sample size (3112 participants) the coronary calcification most occurs in the left anterior descending (44% of total) most commonly affected followed by the right coronary (12%), left circumflex (10%) and left main (6%). These patterns were similar across age, gender, and race/ethnicity. Due this, in the next studies, the statistical analysis will be presented on the sufficient sample sizes that will be related with randomized diagnostic studies, taking into account the demographic data, the prevalence and severity of disease in the study population in accordance with requirements for minimum sample size for analysis of sensitivity and specificity.

Limitations include also technical factors that impact on the accuracy of diagnosis. The most important of these is the image quality, on the basis of which the 3D geometry of the coronary vessels and stenoses is reproduced. Both spatial resolution, temporal resolution, and contrast resolution are all important aspects for CT image quality. Some patient-specific parameters, including body habitus, heart rate and health condition may also impact on the image quality. In case of cardiac imaging the spatial resolution are usually fixed at 512 × 512, minimum layer thickness: < 1 mm, minimum axial resolution: 0.35 mm what in practice allows for segmentation of coronary vessels over 1.5 mm. Additionally, the artefacts of CCTA, such as motion, misalignment, low contrast, or blooming, may impair the diagnostic reliability of VCAST.

The results presented in this papers are based on manual segmentation and reconstruction and may affect diagnostic quality and reproducibility, therefore, three-dimensional anatomic models are manually verified by two independent and experienced image analysis experts.

However, the possible segmentation errors are duplicated in compared models, real and reconstructed, so the impact of these errors on the diagnostic indexes like EFR are minimal. Likewise, the linear losses of energy associated with blood viscosity and associated with the influence of coronary artery geometry (in addition to stenosis geometry) in both models are approximately the same and therefore can be neglected.

The manual segmentation and reconstruction of the coronary arteries are subjective, operator-dependent, prone to errors, and very time-consuming. This is why in the future we plan to use the machine learning algorithm based on deep, convolutional neural network, that does not require any human–computer interaction and significantly reduce the time of 3D models creates.

## Conclusion

The study presented promising results of a non-invasive and comparative of test to support of prevention of coronary disease and functional evaluation of stenosed vessels in patients with ischemic heart disease.

A multicenter studies are required, taking into account the demographic data, the prevalence and severity of disease in the study population in accordance with requirements for minimum sample size to establish diagnostic performance of the test and confirm the relevance and verification of the VCAST indices.

## Supplementary Information

Below is the link to the electronic supplementary material.

Supplementary file1 (PDF 389 kb).

Supplementary file2 (PDF 797 kb).

Supplementary file3 (PDF 1027 kb).

## Data Availability

The datasets used and/or analysed during the current study are available from the corresponding author in the Biocybernetics Laboratory of Prof. Z. Religa Foundation of Cardiac Surgery Development Wolnosci st. 345a; 41-800 Zabrze, Poland, on reasonable request.
