# Supplementary material for: The Comparative Method Based on Coronary Computed Tomography Angiography for Assessing the Hemodynamic Significance of Coronary Artery Stenosis
Source: Cardiovasc Eng Technol. 2023 Mar 3;14(3):364–79. doi: 10.1007/s13239-023-00658-2 (PMC10412489; doi:10.1007/s13239-023-00658-2)
Supplement: Supplementary file 1 — Supplementary file1 (PDF 389 kb). [file 13239_2023_658_MOESM1_ESM.pdf]

APENDIX 1

The principles of Virtual Cardiac Shear Stress are shown on the graph (Fig.1). Increasing flow rate at the inlet to the vessel causes an increase in the inlet energy of flow and in the area of stenosis an increase in disturbance of flow forces. Total energy losses during flow through the coronary vessel can be estimated as the energy difference between inlet of the vessel and the outlet from all branches.

For flow through tree coronary vessels, there is a global pressure loss over the vessel length due to friction and local pressure loss due change of geometries, directly at the site of vessel stenoses, branches or bends. A linear increase in flow rate, during a virtual exercise test, causes an increase in pressure drop, which depends on the shape of the coronary vessels (branching arches), their diameters and the degree of stenosis. In the model with stenosis, this decrease is much higher than in the reconstructed model (Fig.1a).

In general, the pressure in individual branches of both models (with stenosis and reconstructed) can be represented in flow rate function by a quadratic polynomial.

The function is a quadratic polynomial where the first element determines the effect of the viscosity forces and the second one is due to the convective acceleration and inertial forces, which lead to energy losses through flow separation, flow disturbance and turbulence flow [1]. However, for high stenosis degree and high flow rate this relationship shows deviations from this quadratic function [2]. The coefficients of this polynomial can only be determined empirically. During physical exertion, the heart is capable of augmenting coronary blood flow over 5-fold above resting values and value of systolic blood pressure to increase up to 26 kPa [3,4].

Due to the regulation of coronary vascular resistance (vasodilation) the blood flow is maintained relatively constant over a wide range of perfusion pressures. Outside this range, flow becomes pressure-dependent and the loss of autoregulation is observed. In case of loss of autoregulation, the narrowing of the vessel increases the flow resistance and causes an additional energy drop.

In order to confirm this linear relationship a full virtual exercise test was performed for the selected patient. For this reason, during the virtual exercise test, the linearly increasing flow rate was used on the inlet of the reconstructed coronary vessel until a maximum flow of 20 ml/s was reached (maximum

Reynolds number ~5000). The numerical calculation for transient flow was performed for linearly increasing inflow from 0 ml s^{-1} to 20 ml s^{-1}, over 4 s with time step of 0.002 s

For the acceleration of the execution time of test we used a high-performance computer (HPC) . Parallel processing was be performed using 120 CPUs. Average wall-clock time for typical steady flow test of coronary arteries was approximately 540s whereas for the transient flow, this time was over 100 times higher.

VCAST is based on the assumption that under the same flow conditions (coronary autoregulation still working), the pressure relationship between the distal pressure downstream to the stenosis and the pressure at the same site in the reconstructed model is linear. In this case, the resistance of the vessel with the stenoses is reduced due to the additional pressure drop. This makes it possible to calculate the EFR as the ratio of pressure in the stenosed model to pressure in the reconstructed model, for a patient-specific flow rate. However, a linear relationship was found between distal pressure, downstream to the stenosis and the pressure in the same place in the reconstructed model. The angle of the linear relationship corresponds to the EFR value (Fig1c).

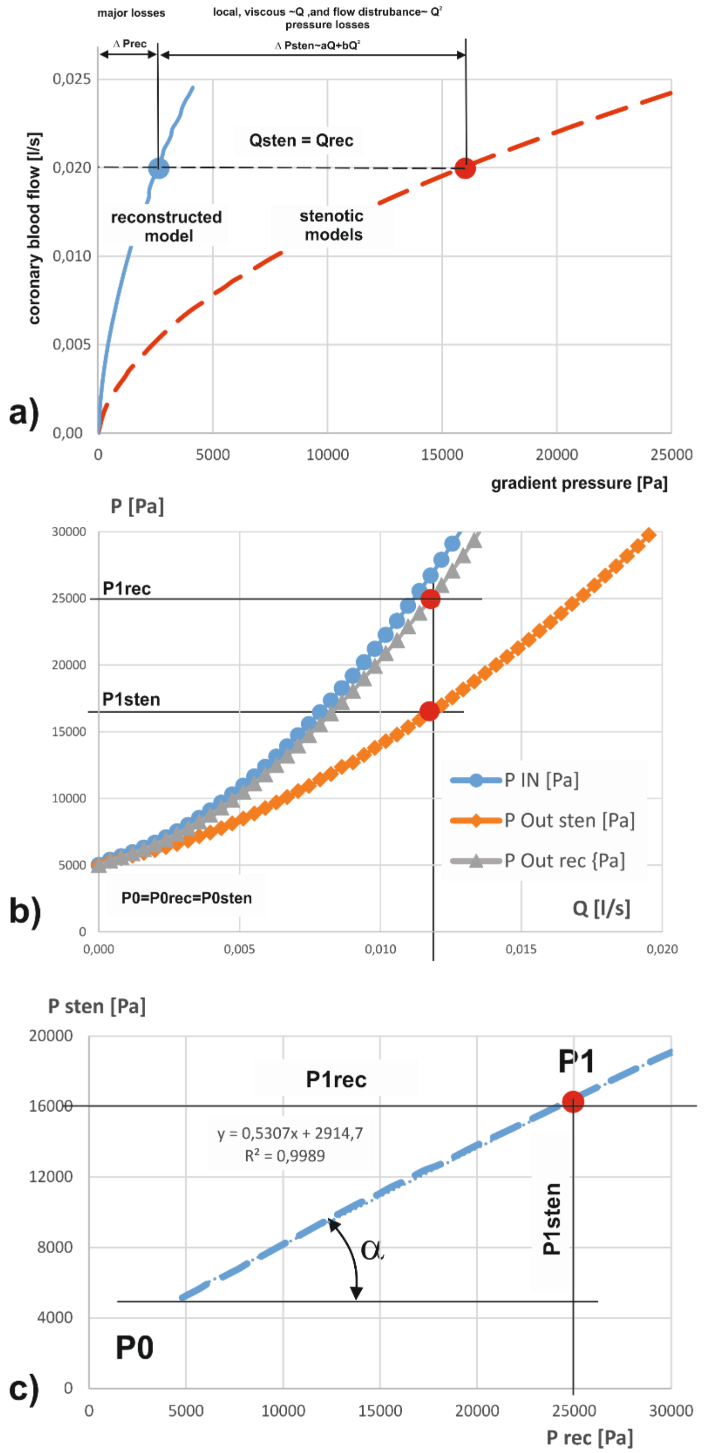

FIGURE 1. Example of virtual exercise test under a linear increase in flow rate at the inlet of the left coronary artery with 72% stenosis in mLAD (mid Left Anterior Descending Artery); a) flow-pressure gradient function showing the change in pressure drop in selected branch with narrowing (red dashed line) and the same branch of the reconstructed model (blue line), b) pressure-flow function showing the changes in pressure on inlet (blue line), in selected branch with narrowing (red line) and the same branch of the reconstructed model (gray line), c) the relationship between pressure downstream

narrowing of stenosed model (Psten) and pressure in the same place of the coronary branch of reconstructed model (Prec). Point P0 corresponds to a pressure value for a flow rate of 0 ml s^{-1} and point P1 corresponds to a pressure value for a flow rate of 20 ml s^{-1}.

References

1. Young DF, Tsai FY. Flow characteristics in models of arterial stenoses. I. Steady flow. J Biomech. 1973;6(4):395-410.
2. Ryu K et al. Importance of accurate geometry in the study of the total cavopulmonary connection: computational simulations and in vitro experiments. Ann Biomed Eng. 2001;29(10):844-53.
3. Duncker DJ, Bache RJ. Regulation of coronary blood flow during exercise. Physiol Rev. 2008;88(3):1009-86.
4. Fletcher GF et al. Exercise Standards for Testing and Training. A Statement for Healthcare Professionals From the American Heart Association. Circulation. 2001;104:1694–1740.
