# Supplementary material for: The Comparative Method Based on Coronary Computed Tomography Angiography for Assessing the Hemodynamic Significance of Coronary Artery Stenosis
Source: Cardiovasc Eng Technol. 2023 Mar 3;14(3):364–79. doi: 10.1007/s13239-023-00658-2 (PMC10412489; doi:10.1007/s13239-023-00658-2)
Supplement: Supplementary file 2 — Supplementary file2 (PDF 797 kb). [file 13239_2023_658_MOESM2_ESM.pdf]

APPENDIX 2

In order to choose the correct model for the entire range of Reynolds number and flow complexity associated with both stenosed and reconstructed coronary arteries, the comparison of laminar and selected turbulence models with experimental data of FDA benchmark nozzle model were used [1], (Appendix 3).

The simulation results were compared with experimental data respectively, for radial velocity at location of 0.032 m and axial velocity and axial pressure, for steady flow with throat Re numbers of 500, 2000 and 3500 respectively.

The simulation results clearly showed dependence on the kind of turbulence model, the place of measurement as well on and Re numbers. They are consistent with the results presented in the paper [2]. It should be noted that the experimental measurements in some measurement areas are characterized by a significant measurement error (the error bars on Fig.1). However, the impact of these errors on the test results is reduced as the values of the VCAST parameters are calculated as the ratio of the pressure averaged over the cross-section of the vessel.

The k-omega SST low Re model was finally selected, which ensured the assumed accuracy of calculation VCAST parameters in validation studies over the whole investigated range of Re.

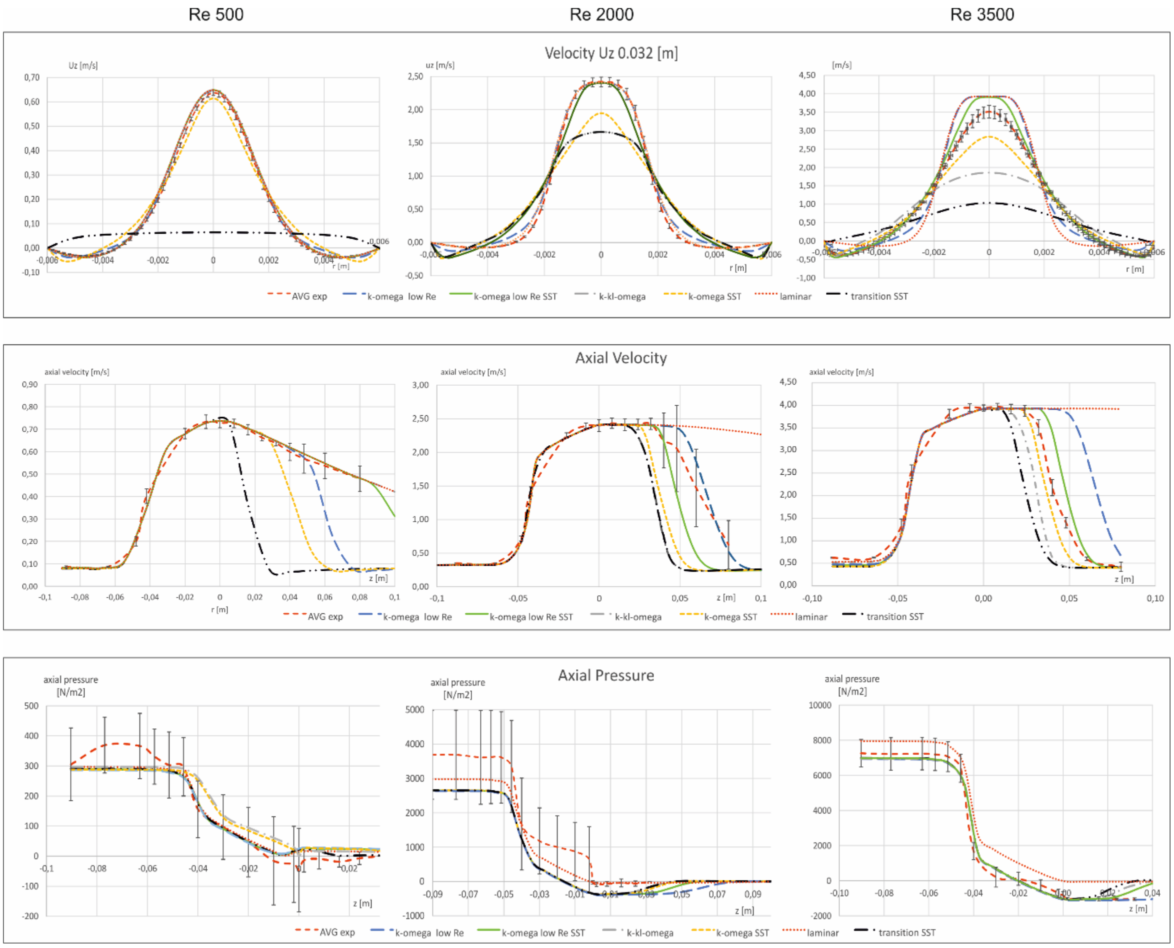

Figure 1 The influence of turbulence models on velocity profile, axial velocity and axial pressure of nozzle FDA model. The comparison of the results obtained from experiments and CFD studies for steady flow with throat Reynolds numbers 500, 2000 and 3500. The error bars represent the experimental uncertainties (U_{exp}).

REFERENCES

1. Hariharan P, D'Souza GA, Horner M. Morrison TM, Malinauskas RA, Myers MR. Use of the FDA nozzle model to illustrate validation techniques in computational fluid dynamics (CFD) simulations. PLoS One. 2017 Jun 8;12(6):e0178749.
2. *Stewart* SFC, et al. Assessment of CFD Performance in Simulations of an Idealized Medical Device: Results of FDA's First Computational Interlaboratory Study. Cardiovascular Engineering and Technology. 2012;3(2).
