# Supplementary material for: The Comparative Method Based on Coronary Computed Tomography Angiography for Assessing the Hemodynamic Significance of Coronary Artery Stenosis
Source: Cardiovasc Eng Technol. 2023 Mar 3;14(3):364–79. doi: 10.1007/s13239-023-00658-2 (PMC10412489; doi:10.1007/s13239-023-00658-2)
Supplement: Supplementary file 3 — Supplementary file3 (PDF 1027 kb). [file 13239_2023_658_MOESM3_ESM.pdf]

SOLUTION VERIFICATION AND NUMERICAL MODEL VALIDATION

To solution verification and validate of numerical model was performed in accordance with ASME V&V 20-2009 Standard for Verification and Validation in Computational Fluid Dynamics and Heat Transfer [1,2].

SOLUTION VERIFICATION

The Grid Convergence Index (GCI) method, based on Richardson extrapolation theory, was adopted for conducting the grid convergence study and for obtaining the numerical uncertainty due to discretization of the flow domain into finite volumes.

This approach requires conducting test runs for three different mesh resolutions. In each run, the control variable is collected in an earlier planned manner. Having described values gathered, the Richardson extrapolation is used in order to estimate the control variable value for mesh spacing equal to zero. Based on the approximated value, grid convergence index is calculated for the control variable. Having GCI calculated, it is finally used to check if the received solution lies in the asymptotic range of calculated extrapolation. The mesh refinement study was performed on a selected patient-specific model of left coronary artery with stenosis (~ 75%) in the proximal segment of the left circumflex (CX) branch (Fig.1). The control variables, characteristic parameters of VCAST (pressure, FFR_{sten}, EFR) were calculated in two planes, normal to vessel axis, behind the stenosis (~ 6D) in the circumflex (CX) and obtuse marginal (OM1) branches.

The numerical uncertainty will be estimated using the GCI formula by using a conservative expansion factor k=1.5:

$$U_{num} = GCI_{fine}^{21}/1.5 = \frac{F_s \times \left|\frac{\varphi_2 - \varphi_1}{\varphi_1}\right|}{r_{21}^p - 1}$$

where: $p = [1/\ln(r_{21})][ln|\varepsilon_{32}/\varepsilon_{21}|+q(p)]$, and ε is the absolute error in the output variable of interest, in case constant refinement factor q(p)=0,

φ – is computed using two subsequent grids (for example, grids #1 & #2), e.g.,

$\varepsilon_{21} = \varphi 2 - \varphi 1$,

Fs - a factor of safety - value will be selected based on previous empirical studies for a three-grid convergence study.

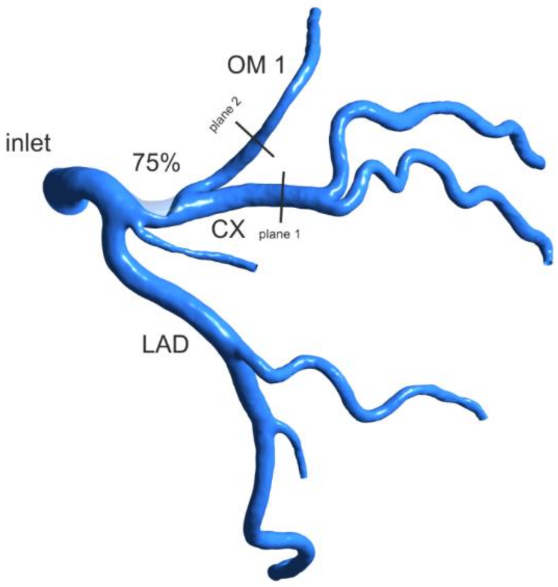

FIGURE 1. The left coronary artery with stenosis (~ 75%) in the proximal segment of the left circumflex (CX) branch

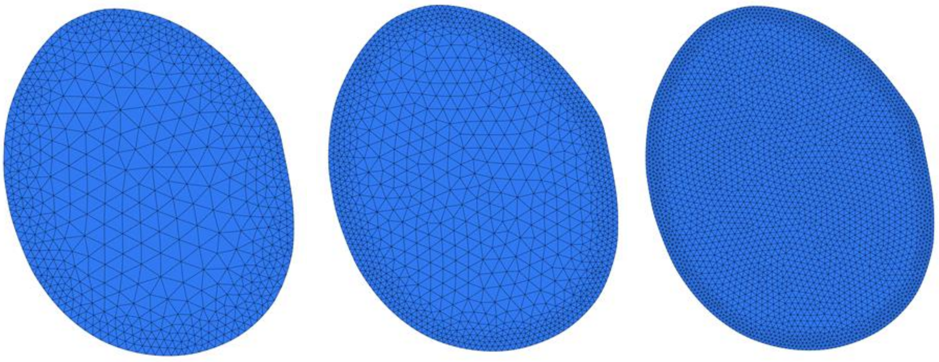

FIGURE 2. Inlet mesh with tetrahedral elements shown and visible refinement in near-wall region for medium grid for Coarse, Medium and Fine grid respectively with grid refinement factors r21 ~r32 ~1.5

The coarse, medium and fine tetragonal mesh, refined in near-wall regions, were used (Fig.2). Three grid sizes were generated using a constant value of refinement factor of ~1.5. The details of grids are provided in Table 1.

grid	number of elements	number of nodes	refinement factor	min/max element
**1** - fine	7913821	1523011		0.08/0.13
**2** - medium	2404919	497839	1.54	0.12/0.3
**3** - coarse	651013	137823	1.49	0.3/0.4

TABLE 1. Details of the grids used for the grid convergence study

The result of the grid convergence study for characteristic sites behind the stenosis are summarized below in tables 2.

OM1 – Pressure plane 2						
**Parameters**	**stenosed**			**reconstructed**		
	**Fine**	**Medium**	**Coarse**	**Fine**	**Medium**	**Coarse**
Pin [Pa]	19220	19300	19290	19220	19300	19290
P plane 2 [Pa] ($\varphi_{i,}$)	14730	14950	15410	17490	17540	17530
Error ε^{21}, ε^{32}		220	460		50	10
**$\|U_{num}\|_{21}/\|U_{num}\|_{32}$**	**0.01321/0,03286**			**0.000577/0.000115**		

**CX – FFR$_{sten}$ plane 1**				**OM 1 – FFR$_{sten}$ plane 2**		
**Parameters**	**Fine**	**Medium**	**Coarse**	**Fine**	**Medium**	**Coarse**
Pin [Pa]	19220	19300	19290	19220	19300	19290
Pout [Pa]	16910	17110	17450	14730	14950	15410
FFR$_{sten}$ ($\varphi_{i.}$)	0.8798	0.8865	0.9046	0.7663	0.7746	0.7988
Error ε^{21}. ε^{32}		0.0067	-0.0180		0.0082	-0.0242
**$\|U_{num}\|_{21}/\|U_{num}\|_{32}$**	**0.01027/0.02746**			**0.01371/0.04001**		
**CX – EFR plane 1**				**OM1 – EFR plane 2**		
Pin [Pa]	18780	18840	18830	14730	14950	15410
Pout [Pa]	16910	17110	17450	16910	17110	17450
FFR$_{sten}$ ($\varphi_{i.}$)	0.8907	0.899	0.9197	0.8421	0.8523	0.8790
Error ε^{21}. ε^{32}		0.00774	0.01853		0.01014	0.02672
**$\|U_{num}\|_{21}/\|U_{num}\|_{32}$**	**0.0132/0.0326**			**0.01656/0.04313**		

TABLE 2. The numerical uncertainty (Unum) for the pressure, FFR$_{sten}$ (Fractional Flow Reserve for stenosis model) and EFR (Energy Flow Reference Index) downstream to the stenosis (plane 1 and plane 2)

NUMERICAL MODEL VALIDATION

The geometry of model to Validation reflects of the severe coronary stenosis with hemodynamic significance. The model includes a gradually converging section, a throat region and a sharp-edged sudden expansion. These geometric features induce complex flow phenomena including adverse pressure gradients, recirculating flow and high and low shear stress zones, which mimic flow features of cardiovascular devices such as those found in the regions of coronary stenosis.

Based on interlaboratory study was defined the FDA reference nozzle model geometry and flow conditions, and identified comparison metrics to determine the suitability and methodology for simulating fluid flow in an idealized medical device, as required by the standard ASME V&V 20 -2009. The model geometry is a 0.012 m diameter cylindrical nozzle with a conical collector and sudden expansion on either side of a 0.04 m long, 0.004 m diameter throat (Fig.3a).

This model very well reflects the hemodynamic properties of turbulent flow through the standard vasoconstriction, so we decided to perform a validation study of our test on the results made by independent laboratories, which are available in the data database;

https://ncihub.org/wiki/FDA_CFD/ComputationalRoundRobin1Nozzle and described in: [2,3,5,6].

The flow by nozzle model corresponds to coronary flow during cardiac stress with significant stenosis of 89% and maximal (throat) Reynolds number of 3000. For this reason, to validate the Virtual Cardiac Stress Test (VCAST methods), the simulation results will be compared with experimental data of nozzle FDA model for throat Re numbers of 2000, and 3500, respectively.

Input parameters for the CFD simulations were determined from an inter-laboratory particle image velocimetry (PIV) study, consisting of 5 datasets from 3 laboratories.

The uncertainties in the numerical simulation and experimental results due to input parameter uncertainties were quantified following the ASME V&V 20 standard. Techniques presented in the ASME V&V 20-2009 standard were applied to estimate:

1. the comparison error (E = |S-D|), which represents the difference between the mean experiment (D) and simulation (S) results, and
2. the validation uncertainty (Uval), which is a summation of the uncertainties in the numerical simulations, input parameters and the experimental data.
3. model error – due to modeling assumptions.

Validation requires comparing the comparison error |E| with the value of the U_{val} validation metric) as the sum of the squared uncertainty of the experimental data Uexp, the uncertainty of the result of the numerical simulation Unum and the uncertainty of the data used as input parameters in the U_{inp} calculations performed. The validation comparison error (discrepancy) id defined as: E=S-D, where: S-simulation solution, D – experimental data.

The validation uncertainty combines the uncertainties due to numerical errors (U_{num}), input parameters (U_{inp}), and experimental measurements (U_{exp}) and will be calculated as:

$$U_{val} = \sqrt{U_{num}^2 + U_{inp}^2 + U_{exp}^2}$$

The modeling error (δ_{model}), which is the error in the simulation due to modeling assumptions and approximations, will be bounded using U_{val} as follows:

$$\delta_{model} \epsilon \; [E - U_{val}, E + U_{val} \;]$$

The Grid Convergence Index (GCI) method, based on Richardson extrapolation theory, was adopted for conducting the grid convergence study and for obtaining the numerical uncertainty due to discretization of the flow domain into finite volumes.

Numerical uncertainty (U_{num}) was estimated from the results of a mesh convergence study consisting of a series of three computational meshes (Coarse, Medium, Fine).

Grid convergence study was conducted in order to obtain a grid-independent solution, minimize the numerical error, and estimate the numerical uncertainty due to finite volume discretization.

The experimental uncertainty will be estimated as the standard deviation of 5 measurement data from made by 3 independent laboratories (Fig.3b). In order to compare the simulation results with the experimental results, at the same measurement points, the interpolation of simulation data in the Matlab (MathWorks®) environment was used.

The U_{exp} for FFR was calculated as mean differentials maximum error of FFR=P/Pin function where P is axial pressure experimental values on the reference level of 16000 Pa with standard deviation of 5 measurement data from made by 3 independent laboratories.

The uncertainty in the quantities of interest (i.e. simulation outputs) due to uncertainty in the simulation input parameters will be estimated using the sensitivity coefficient method for uncertainty propagation. For each case, the overall uncertainty in each simulation output parameter due to uncertainty in the input parameters.

From the experimental measurements, volumetric flow rate Q, dynamic viscosity μ and turbulent intensity **τ**, were selected as the input parameters for the uncertainty analysis.

Each input parameter (Xi) was perturbed above and below its nominal (mean) value by a relative perturbation size, ΔX/X, of 7.5e-04 in order to minimize the truncation and round-off errors [4].

The comprehensive uncertainty analysis, in accordance with the experimental measuring points , along axial and radius of the nozzle, for pressure drop, axial velocity, radial velocity at location of 0.032 m as well FFR was estimated.

A significant modeling error was observed for the pressure gradient, which results from a very large experimental pressure measurement error (Fig.3a) and input error.

However, for VCAST, the most important parameter, on the basis of which the hemodynamic significance of stenosis is assessed, is relative parameter FFR. In this case the $\delta_{model} \epsilon$ [-0.0189, 0.0726] and [-0.0161, 0.0241] for Re=2000 and Re=3500 respectively.

It should be noted that, the estimate of the error caused by the uncertainty of inputs to each model required a perturbation study. The perturbation size affects the result in input uncertainty of each input parameter. The proper selection of perturbation size carefully is very difficult and important. This process highlights the impact of each parameter. If perturbation size is too large, the truncation error, will be too large, but it is too small, machine round off becomes significant [4]. The recommended range is $10^{-9}<\Delta X/X<10^{-3}$. For nozzle model, we use the relative perturbation size, ΔX/X, of 7.5e-04 (near maximal values) according to [30]. In the case of a coronary vessel, the statistical inlet flow rate is approximately 3 times lower than the inlet flow rate of the nozzle model. So, the perturbation size should be smaller, $\sim 10^{-5}$, which may further reduce the validation and numerical uncertainty errors.

For the purpose of computing numerical uncertainties due to absolute error ε, we have selected the following mean values of output variables φ (Tab.3):

- axial pressure drop,
- axial velocity,
- radial velocity in cross-sectional cuts of 0.032 m and
- Fractional Flow Reserve.

The numerical uncertainties U_{num} was calculated for conservative values of k =1.5

$$U_{num} = GCI/k.$$

Since the validation studies are to confirm the correctness of the model selection to assess the significance of coronary stenosis, an important parameter of the study is the Fractional Flow Reserve (FFR) [8]. In this case the FFR is calculated as the ratio of axial pressure (Pd), measured in the nozzle model, at a distance of 0.03 m downstream to the end of the throat to the of the axial pressure (Pin), measured in nozzle model, at a distance of -0.09 m before the end of throat (this is within the maximal experimental range of axial pressure measurement used by independent laboratories);

$$FFR = \left(\frac{P_d}{P_{in}}\right)$$

During physical cardiac stress test, the heart is capable of augmenting coronary blood flow over 5-fold above resting values and maximal value of systolic blood pressure to increase from 16 up to 26 kPa [8, 9]. Since the experimental data contain only the pressure drop along the vessel axis, therefore, in order to estimate the uncertainty of the FFR assessment, we used the minimum reference pressure of 16000 Pa, which enables the assessment of the maximum FFR error. The lower the reference pressure, the greater the FFR value and thus the greater the FFR error, with the same pressure drop.

The flow by nozzle model corresponds to coronary flow during cardiac stress with significant stenosis of 89% and maximal (throat) Reynolds number of 3000. For this reason, to validate the Virtual Cardiac Stress Test (VCAST methods), the simulation results were compared with experimental data of nozzle FDA model for throat Re numbers of 2000, and 3500, respectively.

REFERENCES

1. Coleman. Hugh & Members. Committee. (2009). ASME V&V 20-2009 Standard for Verification and Validation in Computational Fluid Dynamics and Heat Transfer (V&V20 Committee Chair and principal author).
2. Hariharan P, D'Souza GA, Horner M. Morrison TM, Malinauskas RA, Myers MR. Use of the FDA nozzle model to illustrate validation techniques in computational fluid dynamics (CFD) simulations. PLoS One. 2017 Jun 8;12(6):e0178749.

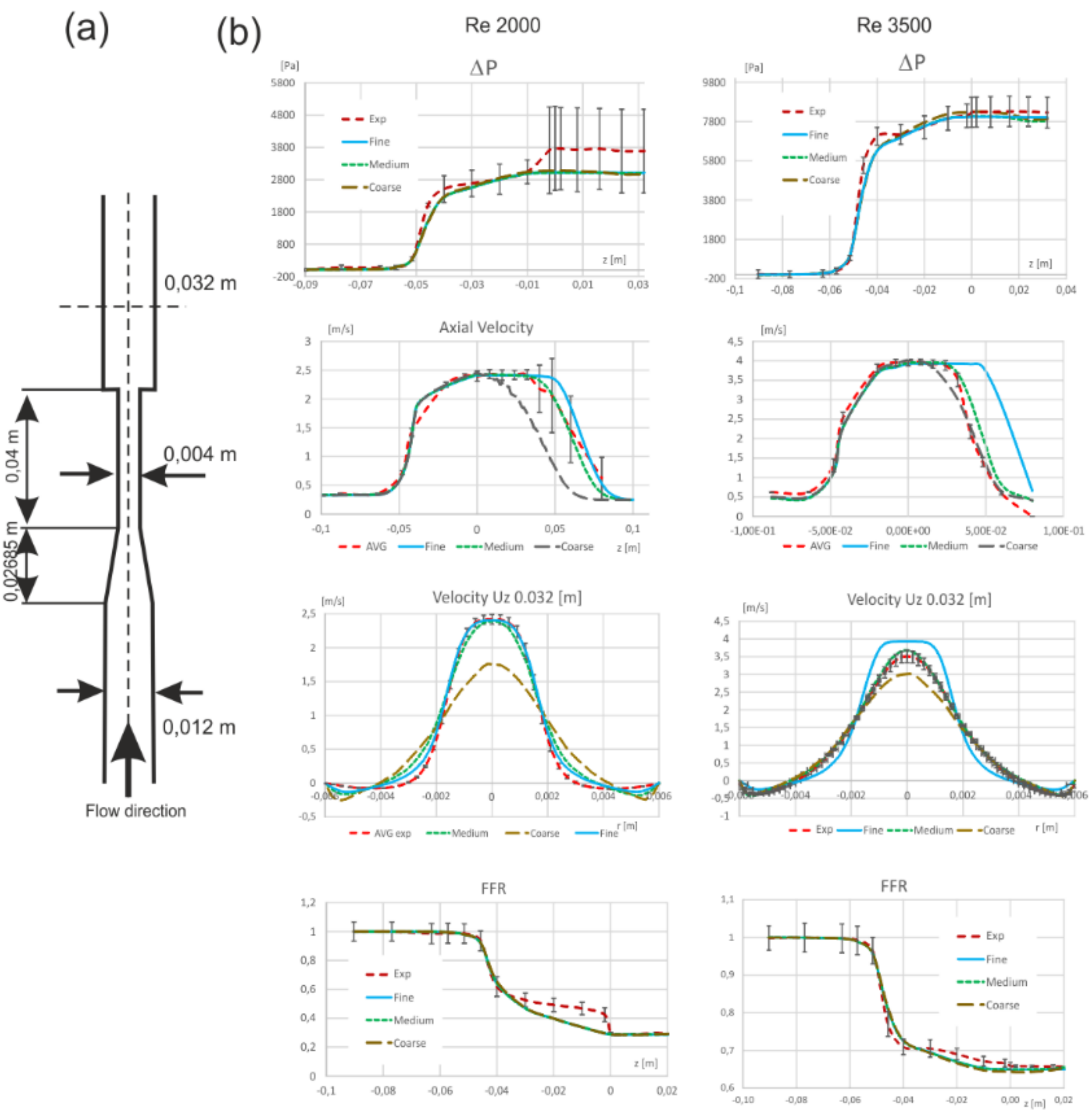

FIGURE 3. Validation of numerical model: (a) Nozzle model based on [30], (b) the experimental and simulation profiles of results of pressure, velocity and FFR in axial and in cross-sectional cuts of 0.032 m for Fine, Medium and Coarse grid and Re=2000 and 3500 respectively. The error bars represent the experimental uncertainties (U_{exp}).

	RE	E=|S-D|	Uval	δ_{model}
**dP [Pa]**	2000	355.322	746.696	[-91.375, 1102.018]
	3500	204.513	615.398	[-10.885, 819.911]
**axial Uz [m]**	2000	0.0368	0.2034	[-0.1666, 0.2402]
	3500	0.4106	0.2875	[0.1230, 0.6982]
**Uz= 0.032 [m]**	2000	0.0311	0.1862	[-0.1551, 0.2173]
	3500	0.1143	0.1343	[-0.0200., 0.2486]
**FFR**	2000	0.0268	0.0457	[-0.0189, 0.0726]
	3500	0.0039	0.0201	[-0.0161, 0.0241]

TABLE 3. The comparison error E, validation uncertainty (U_{val}) and range of modeling error (δ_{model}) for selected output parameters, for Re 2000 and Re 3500

The experimental study

Additionally, the model was validated on the physical mock circulatory cardiovascular system.

The measure of static pressure will be determined at a fixed flow rate, on inlet and branches outlet selected coronary vessel model of patient-specific model of left coronary artery of patients with stenosis (~ 75%) in the proximal segment of the left circumflex (CX) branch.

The simple hydraulic blood circulation system is based on the Windkessel effect (Hwang H, 1984), that simulating the natural blood circulation system by combination of a high-pressure, low-capacity potential energy reservoir, low-pressure and high-capacity volumetric reservoir and elements of peripheral resistance. This system allows precise regulation of flow and pressure parameters (Fig 4).

The system for monitoring and archiving the measured values of pressures and flows enables the simultaneous recording of pressure and flow rate in many channels. It is based on a PC computer equipped with a specialized card of bidirectional analog-to-digital converters and digital signal inputs and outputs. The control program enables the registration of signals, their processing as well as presentation and recording with 100 Hz sampling. The user interface of the program was created in the LabView (National Instrument®) environment.

Flow measurements in individual branches of the coronary model were measured with an OEM Ultrasonic Flow Measuring System, Customized for SW Version with 100Hz Output on CAN and Analogue Interface and SonoTT Clamp-On Transducer. The accuracy of the measurement was ±3.5%. For flow rate below 1 l/min the measurement error is constant of ±0.035 l/min.

Medical pressure sensors DPT 6009 were used to measure the pressure values (measuring range –50 mmHg to +300 mmHg, sensitivity 5 μV / V / mmHg, measurement accuracy +/- 4%).

The measurements were repeated 3 times, after each adjustment of the system. Each series of measurements contained 500 samples obtained within 5 s.

In lab conditions we used blood analog solution in the form of 40% aquatic solution of glycerin to obtain fluid solution density of 1056 kgm^{-3} and dynamic viscosity of 3.5 mPas, which is consistent with the blood values at 37^{0} C and normal hematocrit. Before validation test measurement, each time, the density of the liquid was measured by the aerometer and the dynamic viscosity of the liquid by the calibrated capillary viscometer. Coronary vessel models, both with and without stenosis, were printed in 3D SLA

technology from Accura Clearvue material (Fig.2). The models are varnished on the inside to further smooth the surface of the vessel wall. The thickness of the varnished layer is approx. 0.00005 m. The models were made in a 2:1 scale, therefore research was carried out taking into account the criterion of the similarity of the Reynolds flow (similarity of viscosity forces).

The numerical VCAST™ test was carried out for the same initial and boundary conditions as the experiment (density, viscosity, inlet pressure, outlet flow rates).

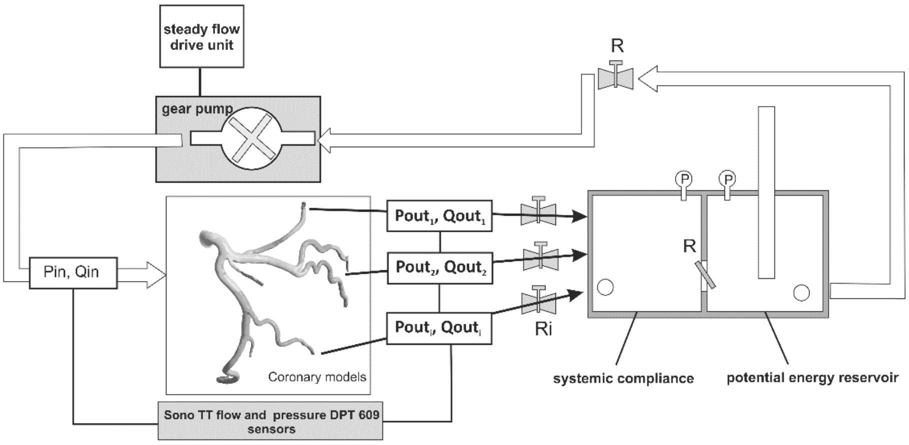

FIGURE 4. The scheme of physical mock circulatory cardiovascular system

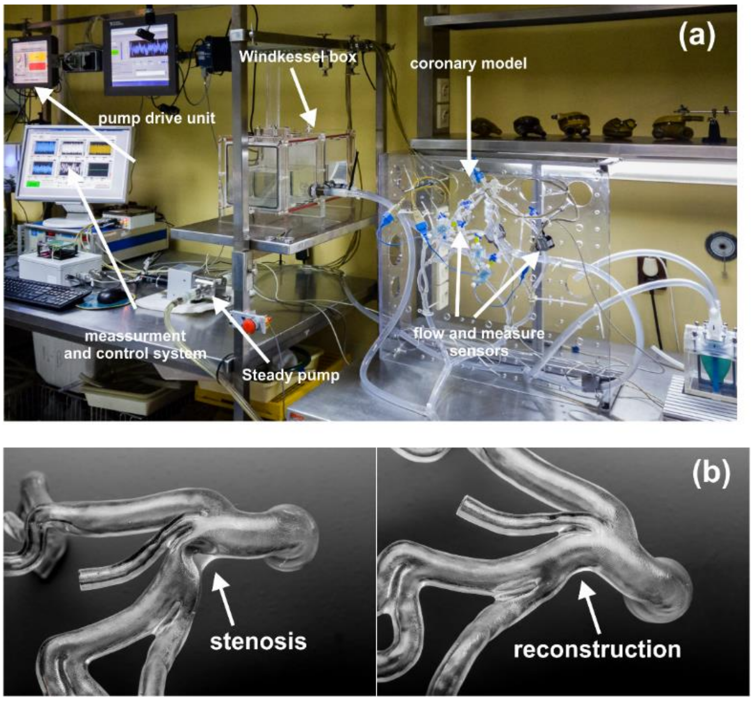

FIGURE 5. a) the physical mock circulatory cardiovascular system, b) 3D printed models of stenosed and reconstructed coronary arteries respectively

The error of FFR and EFR was calculated as mean differentials function of FFR=$(P_i/P_{in})_{sten}$ and EFR= $P_{i\ sten}/P_{i\ rec}$, respectively, where P_i is outlet pressure experimental of selected branches. Maximal error of VCAST indices is 8%.

However, should be noted, the for VCAST test, very important is the set of particular flow rate on the selected outlet of branch. The flow rate in small coronary arteries is below 0.1 l/min. The maximum flow rate measurement error in this range is ±0.035 l / min. Therefore, our coronary model was made in a 2:1 scale. The set flow rates were twice as possible, according to criterion of the similarity of the Reynolds flow, which improved the flow rate accuracy. However, despite this we observed a difficulty in exact settings of coronary flow and keep the right flow balance sheet. The maximum difference between the inlet flow rate and the sum of the outlet flow rate, in the extreme case, was ~ 15%. This affect the measured pressure value because the pressure drop is a quadratic polynomial function of flow rate. More scaling of the model can significantly reduce this error. In addition, lower pressure values were found in some branches without stenosis (Out 4-Out 6) of the stenosed model compared to the reconstructed model, as a result of which the experimental values of FFR_{sten} and EFR in these branches are lowered by about 3%.

It should also be noted that sensors that do not have a calibration certificate issued by an accredited laboratory were used to measure the physical values. The sensors were calibrated only according to internal procedures of Heart Prosthesis Institute of Foundation of Cardiac Surgery Development in Zabrze. The tables and figures below shows the summary of experimental results and comparison with CFD results.

	**Flow rate [l/min]**			
	**CFD**		**experiment**	
	**Q_{sten}**	**Q_{rec}**	**Q_{exp_sten}**	**Q_{exp_rec}**
**IN**	0.516	0.516	0.487±0.034	0.500±0.035
**Out 1**	0.095	0.095	0.098±0.035	0.080±0.035
**Out 2**	0.104	0.104	0.102±0.035	0.109±0.035
**Out 3**	0.063	0.063	0.062±0.035	0.068±0.035
**Out 4**	0.033	0.033	0.036±0.035	0.041±0.035
**Out 5**	0.044	0.044	0.044±0.035	0.048±0.035
**Out 6**	0.035	0.035	0.027±0.035	0.037±0.035
**Out 7**	0.142	0.142	0.141±0.035	0.196±0.035

Table 4. The comparison of the results obtained from experiments and VCAST studies of flow rate

	Static Pressure [kPa]				VCAST indices			
	CFD		experiment		CFD		experiment	
	P_{sten}	P_{rec}	P_{exp_sten}	P_{exp_rec}	FFR_{sten}	EFR	FFR_{exp}	EFR_{exp}
**IN**	19.22	19.22	19.42±0.78	19.59±0.78	1.00	1.00	1.00±0.08	0.99±0.08
**Out 1**	13.40	16.16	13.96±0.56	16.24±0.65	0.70	0.83	0.72±0.05	0.86±0.05
**Out 2**	14.27	16.18	14.32±0.57	16.20±0.65	0.74	0.88	0.74±0.05	0.88±0.05
**Out 3**	14.37	16.17	13.97±0.56	16.11±0.64	0.75	0.89	0.72±0.05	0.87±0.05
**Out 4**	16.13	16.13	15.75±0.63	15.79±0.63	0.84	1.00	0.81±0.06	1.00±0.06
**Out 5**	16.14	16.13	15.74±0.63	16.18±0.65	0.84	1.00	0.81±0.06	0.97±0.06
**Out 6**	16.16	16.16	15.77±0.63	16.14±0.65	0.84	1.00	0.81±0.06	0.98±0.06
**Out 7**	16.15	16.15	16.15±0.65	16.39±0.66	0.84	1.00	0.83±0.06	0.99±0.06

TABLE 5. The comparison of the results obtained from experiments and VCAST™ studies of static pressure and VCAST indices

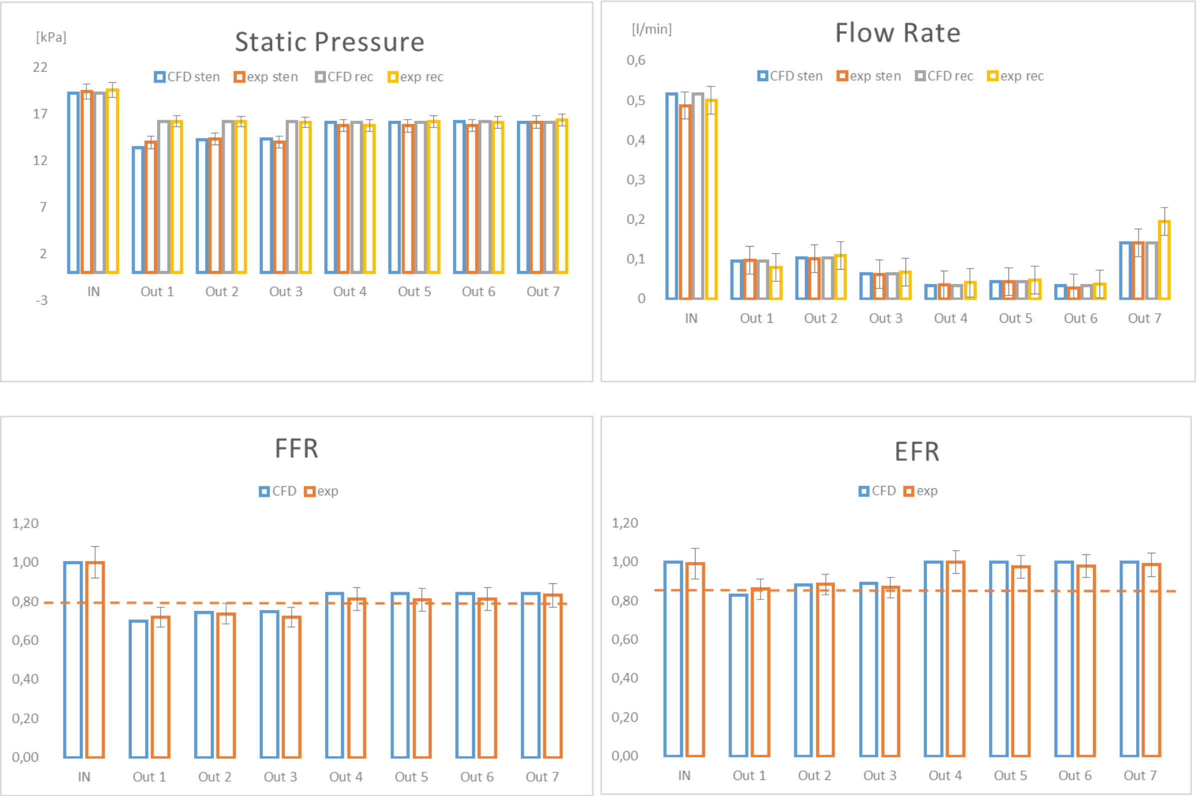

FIGURE 6. The comparison of the results obtained from experiments and CFD studies of static pressure, flow rate, FFR and EFR indices respectively
